# Access to Heterobimetallic M^II^/Cu^I^ Complexes with a Multichelate Platform and Their Reactivity Studies in CO2RR

**DOI:** 10.1021/acs.inorgchem.4c04471

**Published:** 2025-03-03

**Authors:** Samantha L. Peralta-Arriaga, Miguel Ángel Martín-Neri, Carlos García Bellido, Jeremy De Freitas, Sukanta Saha, Francisco José Fernández-de-Córdova, Marc Robert, Orestes Rivada-Wheelaghan

**Affiliations:** † Laboratoire d’Electrochimie Moléculaire, CNRS, 555089 Université Paris Cité, F-75006 Paris, France; ‡ CNRS, Institut Parisien de Chimie Moléculaire, IPCM, 27063 Sorbonne Université, F-75005 Paris, France; § Instituto de Investigaciones Químicas (IIQ), Departamento de química Inorgánica, 201454 Universidad de Sevilla, Avenida Américo Vespucio 49, 41092 Sevilla, Spain; ∥ Institut Universitaire de France (IUF), F-75005 Paris, France

## Abstract

We describe the selective formation of heterobimetallic complexes, exploiting the coordination trends of the developed bis-terpyridyl *trans*-1,2-cyclohexadiamine platform (**L**). Following a stepwise addition, we first reacted ligand **L** toward tetrakisacetonitrile transition metal precursors, [M­(MeCN)_4_]­[BF_4_]_2_ (where M = Fe or Ni), to generate the monometallic complexes **1** ([FeL]­[BF_4_]_2_) and **2** ([NiL]­[BF_4_]_2_). These species were later combined with the tetrakisacetonitrile precursor [Cu­(MeCN)_4_]­[BF_4_], generating the corresponding heterobimetallic complexes **3** ([FeCuL­(MeCN)_2_]­[BF_4_]_3_) and **4** ([NiCuL­(MeCN)_2_]­[BF_4_]_3_). The four species obtained, in high yields, have been structurally characterized. Their cyclic voltammetry analysis revealed the impact of the Cu^I^-atom presence on the heterobimetallic complexes under argon and carbon dioxide (CO_2_) atmospheres. Controlled potential electrolysis studies revealed the instability of complexes **1**–**4** toward CO2RR, generating the heterogeneous material in solution and on the electrode surface. In contrast, CO_2_ photoreduction studies revealed higher stability and photocatalytic activity for the Fe^II^-based complexes (**1** and **3**), generating CO with 88% selectivity.

## Graphical abstract

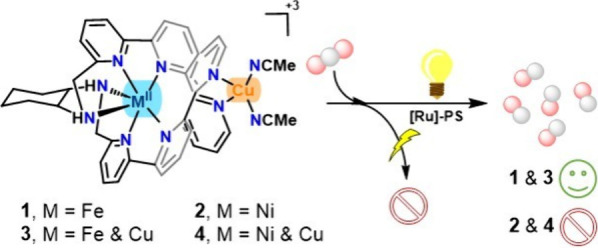

## Introduction

The constant development of spectroscopic techniques for studying protein-derived cofactors has increased our understanding of the structures of multi- and bimetallic molecular complexes found in nature.^1–3^ Over the past decades, this has positively contributed to the increase in the number of synthetic coordination complexes bearing more than one metal.^4–6^ From a synthetic perspective, the selective formation of heterobimetallic complexes requires a ligand platform with selective binding toward the chosen metal precursor.^7^ Many research groups have accomplished this by creating platforms bearing a symmetrical bridging fragment capped by unsymmetrical units, Figure 1a.^8,9^ While others have conceived unsymmetrical bridging units with a common linker that leverages the trends of the ligand first to encapsulate a metal precursor, leaving free donor atoms to later bind selectively to a differentiated metal center, Figure 1b.^10–12^ The development of bimetallic species tends to seek cooperativity between metals:^13,14^ either to modulate the physical–chemistry properties of another metal center,^15–17^ or to activate and transform chemical bonds.^18–20^ Thus, it is not surprising that vast reports on bimetallic cooperation relate to catalytic processes, including molecular electrocatalytic works based on bimetallic systems aiming toward energy storage applications.^21–24^ In this regard, the field of electrochemical Carbon Dioxide Reduction Reaction (eCO2RR) has been experiencing a surge in reports concerning such cooperativity.^24,25^ Although metal–metal interaction has been rarely reported during molecular bimetallic electrocatalytic reduction of CO_2_, cooperativity arising from bimetallic substrate activation has been observed in rare cases.^24,26,27^ Therefore, the development of new ligands,^28,29^ including those specifically targeted for bimetallic cooperativity to promote CO_2_ reduction, remains topical.^24,30,31^

**Figure 1:**
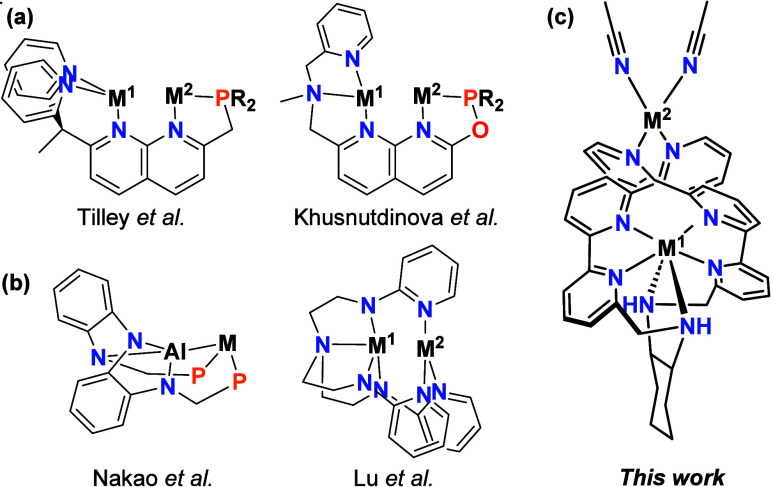
(a) Symmetrical bridging fragment capped by unsymmetrical units; (b) Unsymmetrical bridging units with a common linker; (c) Symmetrical bridging units with a common Linker.

We recently reported the synthesis and characterization of a biscobalt complex stabilized by a multichelate bis-terpyridine pyrazole-bridged ligand that electrocatalytically reduces carbon dioxide (CO_2_) with up to 94% selectivity toward carbon monoxide (CO) at −1.35 V vs Saturated Calomel Electrode in dimethylformamide (0.39 V overpotential).^32^ Encouraged by these results, we continued exploring new bimetallic complexes aiming to reduce CO_2_. Consequently, we synthesize a ligand bearing terpyridine-based coordinating groups,^33^ knowing the advantages such fragments can provide during the CO_2_ electroreduction process.^34^ While modifying the core ligand structure, we aim to achieve different coordination modes that could favor the formation of heterobimetallic compounds.^7^ Thus, we report the synthesis and characterization of ligand **L**, formed by the symmetrical linker *trans-*1,2-cyclohexadiamine, substituted with [2,2′:6′,2″-terpyridin]-6-ylmethylenes at the N-atoms (Figure 1c). The ligand proves effective in selectively generating heterobimetallic complexes through a stepwise addition of transition metal precursor. Hence, we synthesize a set of mono- and heterobimetallic species and examine and compare their structural differences and the effects on the electronic properties of the encapsulated M^II^ metal center in the presence of an added Cu^I^-atom using cyclic voltammetry (CV). Additionally, we analyze the impact on their electrochemical performance under CO_2_ and their stability under photocatalytic conditions toward CO_2_ reduction, following similar strategies to those previously reported.^19,24,35–38^

## Results and Discussion

### Ligand Synthesis and Complexation with Tetrakis Acetonitrile Transition Metal Precursors

Starting from commercially available terpyridine and following our previous report,^32^ we synthesize 6-Methyl-2,2′:6′,2″-terpyridine carboxylate. This compound was later derivatized to form the corresponding [2,2′:6′,2″-terpyridine]-6-carbaldehyde,^39,40^ which is then combined in a ratio of 2:1 with *trans*-1,2-cyclohexanediamine and reduced^41^ to generate the ligand **L**. The ligand **L** was characterized by UV–vis, NMR and HRMS. Combination of **L** with the corresponding tetrakisacetonitrile transition metal precursors, [M­(MeCN)_4_]­[BF_4_]_2_, where M = Fe or Ni, in MeCN at room temperature generates complexes **1**, [FeL]­[BF_4_]_2_, and **2**, [NiL]­[BF_4_]_2_, as shown in Scheme 1. These reactions generate the corresponding monometallic complexes within minutes in high yields under an inert atmosphere. Both complexes are characterized by elemental analysis and HRMS.

**Scheme 1:**
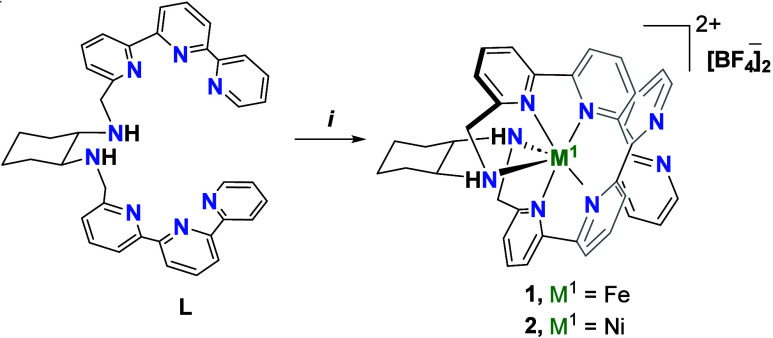
Formation of Monometallic Complexes **1** and **2** [Fn sch1-fn1]

a
*i* = [M­(MeCN)_4_]­[BF_4_]_2_, (M = Fe or Ni), MeCN, room *T*.

The UV–vis absorption spectra of the metal complexes **1** and **2** in MeCN remain dominated by π → π* intraligand excitations (see Figures S5 and S8). Both complexes have paramagnetic qualities in acetonitrile solutions. Their magnetic moments (μ_eff_ **^1^** = 4.55, μ_eff_^**2**^ = 2.67) were obtained in solution measured by the Evans method, and their values fall slightly below the expected range for S = 4 (**1**) and S = 1 (**2**) octahedral complex. In this regard, we could obtain suitable crystals of complex **2** for their single-crystal X-ray diffraction characterization studies. As it is shown in Figure 2, the Ni^II^-atom has an octahedral environment and is bound to 6 out of 8 available N-atoms: 2 N-atoms belong to the *trans*-1,2-cyclohexadiamine (the common linker), and 4 N-atoms belong to 4 pyridyl groups from the two terpyridyl fragments. This arrangement leaves two pyridyl groups available for further coordination with other metal centers. From the magnetic moment and UV–vis spectra we assumed that the iron complex, **1**, should have a similar coordination structure to **2**.

**Figure 2:**
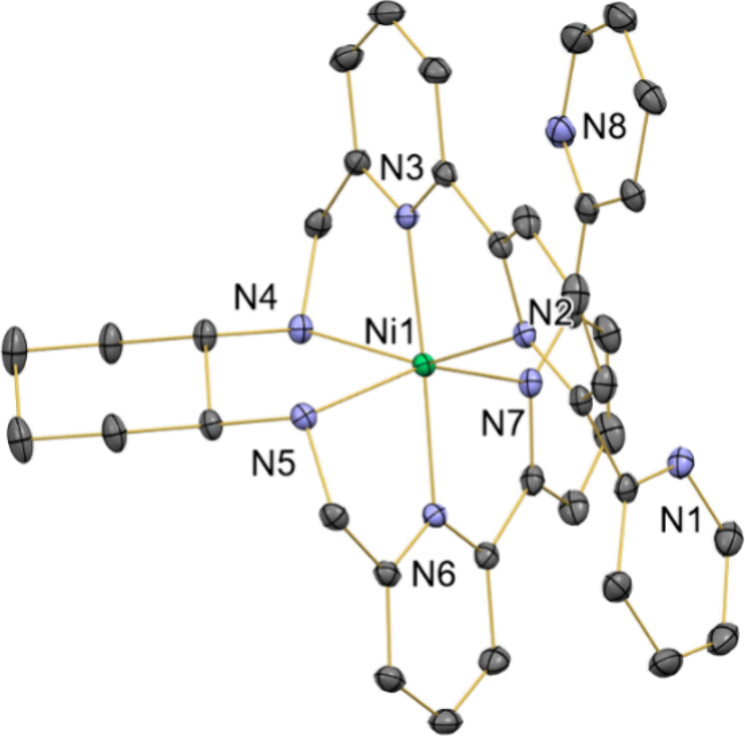
X-ray crystal structures of complex **2** with thermal ellipsoids set at 50% probability. Counterions, solvent molecules and hydrogen atoms are omitted. Selected bond lengths (Angstroms) and angles (degrees): Ni1–N2 = 2.19(2), Ni1–N3 = 1.99(2), Ni1–N4 = 2.14(2), Ni1–N5 = 2.14(2), Ni1–N6 = 2.00(2), Ni1–N7 = 2.20(2); N4–Ni1–N2 = 153.23(6) and N6–Ni1–N3 = 175.98(7).

To form the desired heterobimetallic complexes, we added an equivalent of Cu^I^ precursor to the monometallic species under an Ar atmosphere in acetonitrile. The addition of the copper precursor, [Cu­(MeCN)_4_]­[BF_4_], to **1** and/or **2** at room temperature generated complexes **3**, [FeCuL­(MeCN)_2_]­[BF_4_]_3_, and **4**, [NiCuL­(MeCN)_2_]­[BF_4_]_3_, respectively, as described in Scheme 2, with high yields (>95% isolated). Both heterobimetallic species (**3** and **4**) have been characterized by elemental analysis, and the tricationic species could not be identified by HRMS analysis. This last analysis could indicate the lability of the Cu^I^-atom in the complexes formed.

**Scheme 2:**
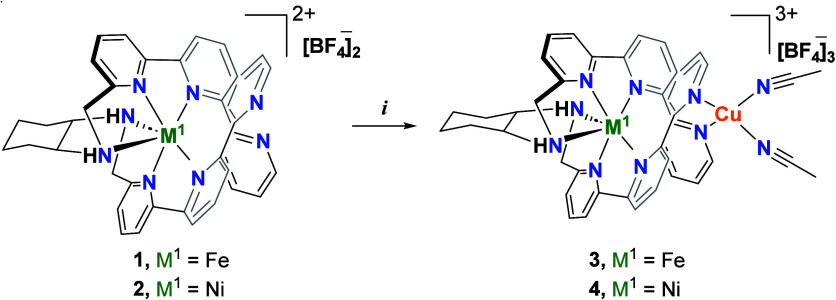
Formation of Heterobimetallic Complexes **3** and **4** [Fn sch2-fn1]

a
*i* = [Cu­(MeCN)_4_]­[BF_4_], MeCN, at room temperature.

Their UV–vis absorption spectra revealed that the coordination of Cu^I^ induces hypochromic shift at specific bands in **3** (280 nm; 22,520 L mol^–1^ cm^–1^) and **4** (315 nm; 24,758 L mol^–1^ cm^–1^) when compared to non-Cu^I^ containing complexes **1** (280 nm; 30,026 L mol^–1^ cm^–1^) and **2** (310 nm; 28,081 L mol^–1^ cm^–1^), see Figure 3. As expected, when we measured their magnetic moments (μ_eff_ **^3^** = 4.43, μ_eff_ **^4^** = 2.67), the obtained values were similar to the monometallic counterparts. This indicates that adding the Cu^I^ has no effect on the paramagnetism of the Fe^II^ and Ni^II^ centers.

**Figure 3:**
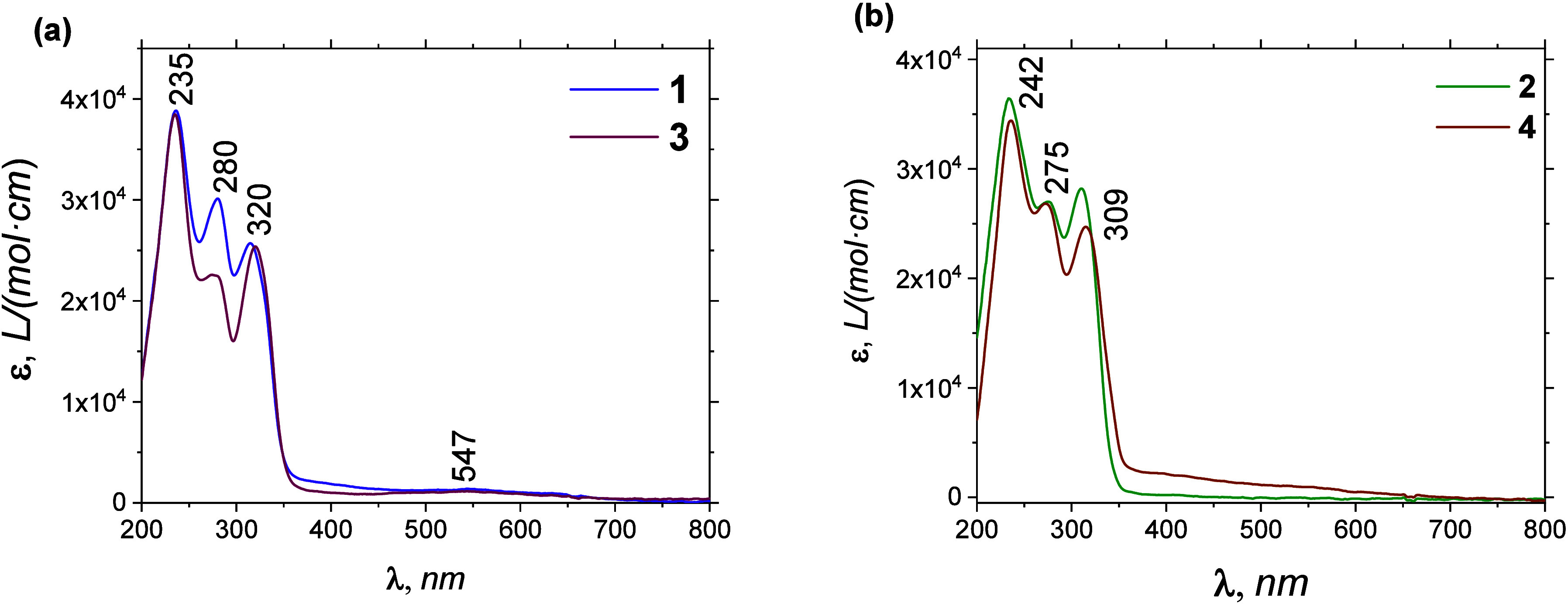
UV–visible spectra comparison of complex **1**–**3** (a) and **2**–**4** (b) in CH_3_CN, using a quartz cell with 1 cm path length and complex concentration of 40 μM.

Complexes **3** and **4** crystallize in mixtures of MeCN/toluene, generating suitable crystals for single-crystal X-ray diffraction studies, Figure 4. Both complexes exhibit similar structures, in which the Cu-center is attached to two pyridyl fragments and two acetonitrile solvent molecules, with the other metal atom being encapsulated within the ligand platform. The introduction of the copper atom in complex **2** does not affect the coordination environment of the Ni^II^-center when comparing bond distances and angles between **2** and **4**. In both complexes (**3** and **4**), the Cu^I^-atom coordination environment is almost identical. However, the M^II^···Cu distance is slightly shorter in complex **3** (3.84 Å) than in complex **4** (3.99 Å).

**Figure 4:**
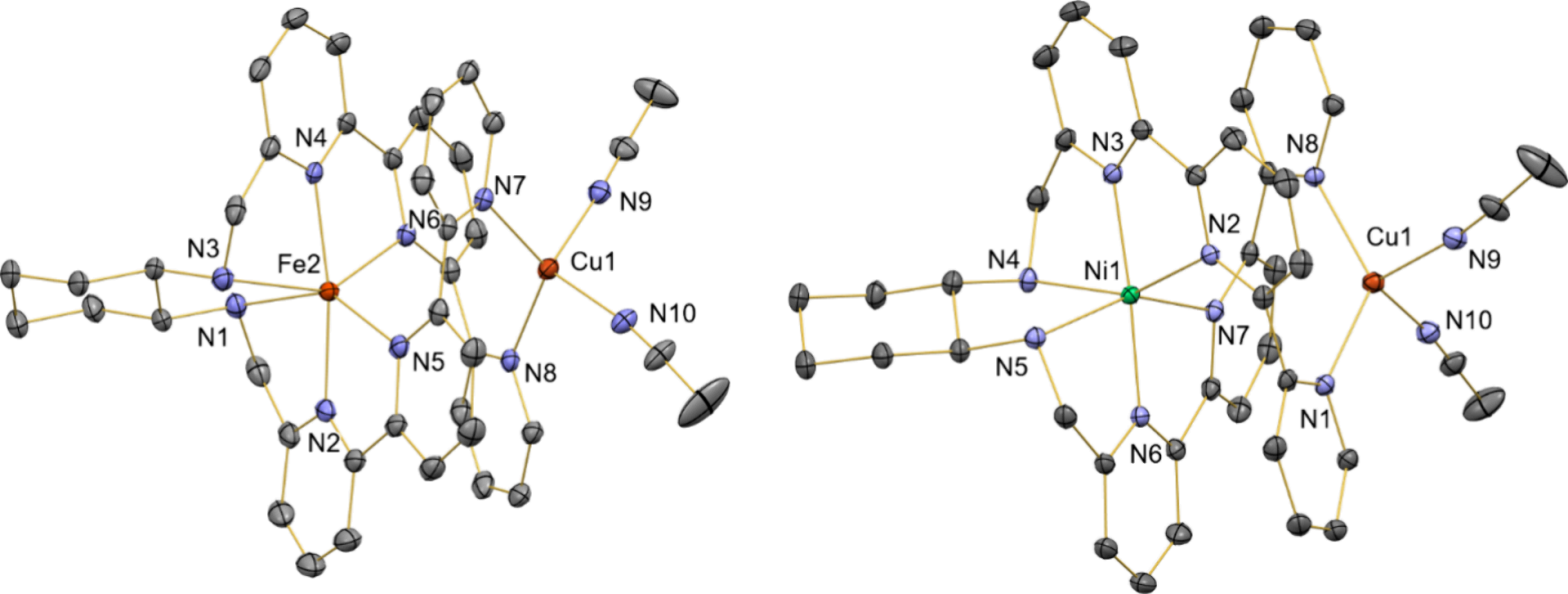
X-ray crystal structures of complexes **3** (left) and **4** (right) with thermal ellipsoids set at 50% probability. Counterions, solvent molecules and hydrogen atoms are omitted. Selected bond lengths (Angstroms). Complex **3**: Cu1–N7 = 2.07(2), Cu1–N8 = 2.08(2), Cu1–N9 = 1.99(2), Cu1–N10 = 2.02(2), Fe2–N1 = 2.34(2), Fe2–N2 = 2.10(2), Fe2–N3 = 2.25(2), Fe2–N4 = 2.09(2), Fe2–N5 = 2.23(2), Fe2–N5 = 2.25(2); Complex **4**: Cu1–N1 = 2.07(2), Cu1–N8 = 2.07(2), Cu1–N9 = 2.01(2), Cu1–N10 = 1.99(2), Ni1–N7 = 2.20(2), Ni1–N2 = 2.20(2), Ni1–N3 = 2.01(3), Ni1–N4 = 2.15(2), Ni1–N5 = 2.17(2), Ni1–N6 = 1.99(3).

### Redox Behavior of Mono- and Heterobimetallic Complexes

We analyzed the redox properties of the four species by cyclic voltammetry (Figure 5). The electrochemical measurements were performed in a dry MeCN solution with 0.1 M Bu_4_NPF_6_ as an electrolyte, using a glassy carbon (GC) working electrode, a Pt-counter electrode (CE), and a saturated calomel electrode (SCE) as a reference electrode (at constant *T* = 293 K) at 0.1 V s^–1^. Under these conditions, CV analysis under Ar of monometallic Fe^II^ complex **1** (Figure 5, red graph) shows a single redox process at −1.32 V vs SCE composed of two consecutive reversible one-electron processes. On the other hand, the monometallic Ni^II^ complex **2** exhibits an irreversible reduction process at −1.24 V vs SCE and a one-electron reversible reduction process at −1.70 V vs SCE (Figure 5, black graph). CV analysis of heterobimetallic complexes containing Fe (**3**) and Ni (**4**) determined that the presence of the Cu^I^-atom has a similar impact on their redox behavior. None of the redox processes already present in the monometallic species seems to be significantly affected by the presence of the Cu center. However, both of the bimetallic species exhibit a new irreversible cathodic wave at different potentials, −0.70 V for **3** and −0.76 V for **4** vs SCE, which has been assigned to the Cu^I^/Cu^0^ reduction process. Furthermore, a new sharp anodic process is observed at −0.2 V vs SCE, probably due to the re-oxidation of surface Cu aggregates, indicative of the demetalation of the Cu center upon reduction (see SI). Knowing the lability of Cu^I^-centers in acetonitrile solutions, we performed the CV of the Cu-precursor, [Cu­(MeCN)_4_]­[BF_4_], under the same conditions. The CV exhibits an irreversible cathodic event at −0.9 V vs. SCE, and an anodic process at 0.15 V vs. SCE. Thus, this result indicates that the Cu^I^-atom remains bound to the ligand in complexes **3** and **4** (Figure S22) once in solution, prior to its reduction.

**Figure 5:**
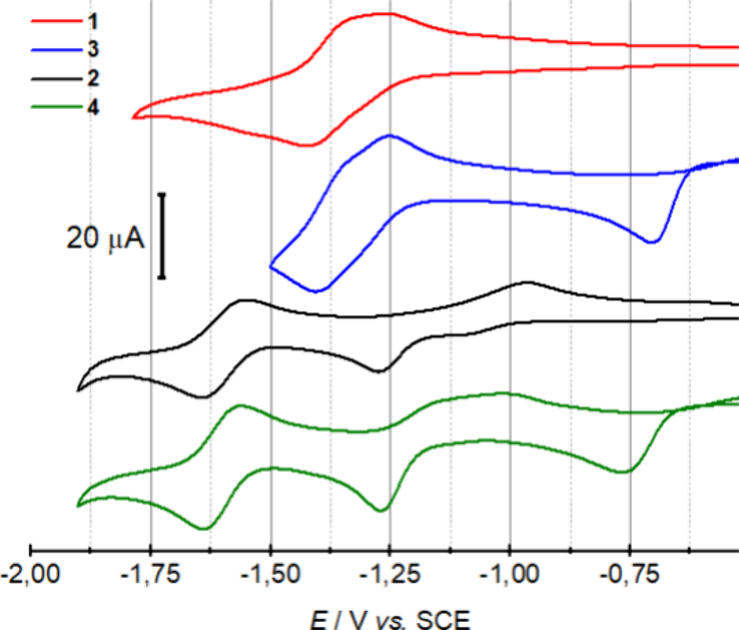
Cyclic voltammetry recorded under Ar, in 0.1 M Bu_4_NPF_6_ dry acetonitrile solutions of complexes **1**–**4** (1 mM) at 0.1 V s^–1^.

### Response of Mono- and Heterobimetallic Complexes toward Carbon Dioxide Reduction Reaction

We have studied the catalytic response of complexes **1**–**4** toward CO_2_ reduction (CO2RR).^42^ Cyclic voltammetry experiments were recorded under a saturated CO_2_ atmosphere, Figure 6a–d, red graph. Even if the response to CO_2_ saturation of the solutions was not uniform among all the systems under study, similarities were found considering the nature of the metal center. Complexes **1** and **3** (with a Fe center) exhibited a similar response, with complex **3** generating more current and exhibiting slightly lower overpotentials (few tens of mV); see the red graph in Figure 6a,c. The electrochemical responses for the complexes containing a Ni center are disparate. Observing higher current enhancement for complex **4**, with almost negligible activity for the monometallic species **2**, see the red graph in Figure 6b,d. Interestingly, the anodic demetalation process observed for complexes **3** and **4** under Ar is drastically reduced in the presence of CO_2_. Furthermore, when a proton source (trifluoroethanol, TFE) was added,^43^ a marked difference is observed between the mono- and bimetallic species. A drastic current increase is observed for complex **3** in the presence of CO_2_, Figure 6c.

**Figure 6:**
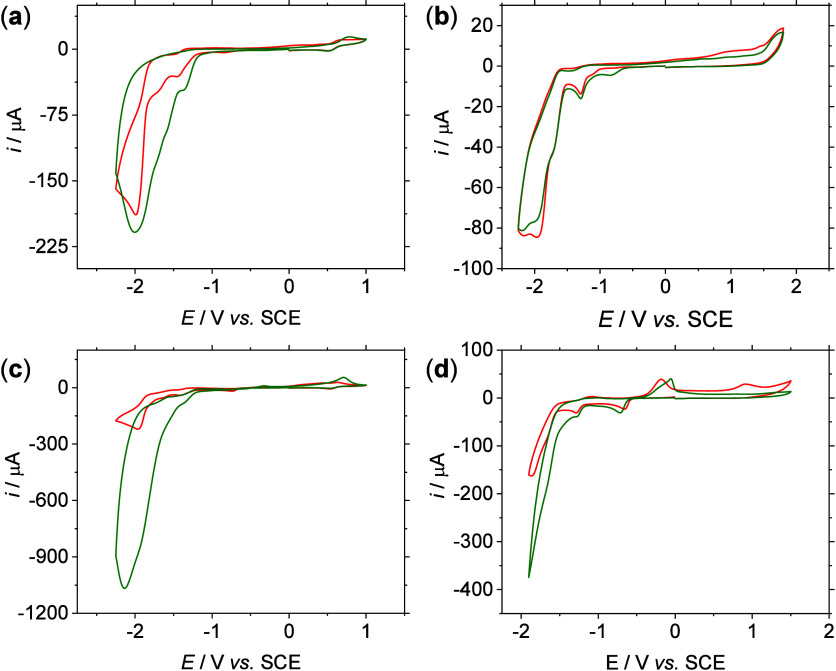
Cyclic voltammetry recorded in 0.1 M Bu_4_NPF_6_ dry acetonitrile solutions under CO_2_ (red) and CO_2_ in the presence of TFE (green) of complexes **1** (a), **2** (b), **3** (c) and **4** (d) (1 mM) at 0.1 V s^–1^.

To further investigate the electrochemical activity of these complexes toward CO2RR, 3h CPE experiments were performed for complexes **1**–**4** (2 mM) in 0.5 M Bu_4_NPF_6_ dry acetonitrile solutions containing TFE (10% V/V) under CO_2_ atmosphere. The results of the different CPEs are presented in Table 1. The low Faradaic efficiencies (FE) obtained for the generation of the different gases are likely due to complex decomposition and the formation of visible heterogeneous material.^44^ In this regard, complex decomposition during CO_2_ electrochemical reduction involves ligand derivatization, as previously reported for terpyridine-,^45^ and polypyridine-based complexes,^46,47^ during electrochemical CO2RR.

**Table 1:** Results from the 3 h CPEs Performed at −1.7 V vs Ag/Ag^+^ with 2 mM Solutions of the Different Complexes

entry	cat.	FE%–H_2_ (μmol)	FE%–CO (μmol)	FE%–CH_4_ (μmol)
1	**1**	1 (0,3)	19 (9)	0 (0)
2	**2**	0 (0)	0 (0,5)	9 (6)
3	**3**	6 (16)	5 (13)	33 (22)
4	**4**	0	0	14 (2)

All complexes (**1**–**4**) were unstable under the CPE conditions employed, with a drastic current reduction of the different species after the CPE experiments, in agreement with their depletion in solution. Only complexes bearing iron (**1** and **3**) generate meaningful amounts of CO. In the case of the Ni-based complexes (2 and 4), deactivation processes were faster when negligent activity was observed (Tables S4 and S6, pages S39–S40 and S43–S44). When using complex **3**, significant amounts of methane (CH_4_) were generated. However, no labeled ^13^CH_4_ (*m*/*z* = 17) is detected during isotope labeling experiments under a ^13^CO_2_ atmosphere, indicating that the methane originates from the solvent (CH_3_CN) (Table S9, Figure S38 and pages S49–S50). X-ray photoelectron spectroscopy (XPS) ex-situ analysis of the glassy carbon plate after the CPEs confirmed the presence of Cu oxide (Figure S39 and page S50). Furthermore, glassy carbon plates subjected to CV studies in a blank solution after the CPE produced higher currents than the initial state (see Tables S4–S8 and pages S37–S48). All these results suggest the involvement of the Cu-based heterogeneous material during the reduction of CO_2_ under electrochemical conditions.^48^

After confirming the instability of these species under electrochemical conditions, we evaluate and compare the response of the different complexes toward CO2RR in photocatalytic experiments, where experimental conditions are significantly different from electrochemical conditions.^49^ Thus, photocatalytic experiments were performed in a quartz cuvette with a 1 cm width pathway (6 mL total vol. capacity) sealed with a rubber septum. All samples were prepared inside a glovebox in a 20 μM solution of the catalysts **1** to **4** in dry acetonitrile (3 mL), using 1,3-dimethyl-2-phenyl-2,3-dihydro-1*H*-benzo­[d]­imidazole (BIH) as the sacrificial electron donor (SED, 25 μM), [Ru­(phen)_3_Cl_2_] (0.2 mM) as photosensitizer and TFE (10% V/V) as the proton source under an Ar atmosphere. Prior to the photocatalytic experiments, the solution was flushed with CO_2_ for 20 min to ensure saturation. One sun irradiation intensity was used, previously measured using a 420 nm filter. After 24 h of irradiation, we analyzed the product by sampling from the headspace (4.5 mL vol) and analyzing the results by GC. CO2RR photocatalytic experiments showed 88% selectivity toward CO generation for complexes **1** and **3**, exhibiting similar catalytic activity observed for complex **1** (131 TON of CO and 18 TON of H_2_) and its heterobimetallic counterpart, complex **3** (152 TON of CO and 20 TON of H_2_). During the photocatalytic process, no formation of microparticles was observed, in contrast to the CPE experiments where heterogeneous material was generated. Additionally, no methane formation was detected during the photocatalytic tests. In contrast, the Ni complexes exhibited extremely low photocatalytic activity, generating negligible amounts of H_2_ and CO (see Table S10 and page S51). Similar differences in the catalytic activity between Ni^II^ and Fe^II^ complexes stabilized with polypyridine ligands have been previously described.^50^

## Conclusions

In summary, we report the synthesis and characterization of a new ligand, **L** (bis-terpyridyl trans-1,2-cyclohexadiamine), and its reactivity toward [M­(MeCN)_4_]­[BF_4_]_2_ precursors (M = Fe or Ni). Monometallic complexes are formed selectively in high yields, and their combination with 1 equiv of [Cu­(MeCN)_4_]­[BF_4_] results in the quantitative and selective generation of heterobimetallic species with the formula [MCu­(L)­(MeCN)_2_]­[BF_4_]_3_. All these complexes have been structurally characterized, and their CVs have been studied under inert and CO_2_ atmospheres. The different complexes (**1**–**4**) showed instability during CPE under a CO_2_ atmosphere with 10% TFE (v/v), resulting in low electrocatalytic activity. Gas analysis from isotope labeling experiments using ^13^CO_2_ detected ^12^CH_4_, indicating that methane formation originates from the solvent. Ex-situ XPS analysis confirmed the presence of Cu-based heterogeneous material formed on the working electrode during CPEs with complex **3**. While the Ni-based complexes (**2** and **4**) did not exhibit any catalytic activity, complexes **1** and **3** exhibited similar photocatalytic activity toward CO2RR, with 88% selectivity toward CO. The comparative CVs analysis for complexes **1**–**4** under an Ar atmosphere indicates that the Cu^I^-atom remains bound to the ligand in complexes **3** and **4** prior to its reduction. However, the results from the CPEs and photocatalytic experiments indicate that the Cu^I^-center in complexes **3** (and **4**) becomes labile during CO2RR. Cu-oxides were observed at the working electrode surface after CPE, with both the monometallic (**1)** and heterobimetallic (**3**) complexes exhibiting the same selectivity and similar photocatalytic activity toward CO2RR. Currently, our new ligand is under investigation with other transition metal centers, so as to trigger and exploit metal–metal cooperativity.

## Experimental Section

### General Considerations

All manipulations, unless stated otherwise, were performed using Schlenk or glovebox techniques under dry argon or nitrogen atmosphere, respectively. THF, toluene, dichloromethane and acetonitrile were freshly distilled prior to use and stored under a nitrogen atmosphere over molecular sieves (4 Å). Diethyl ether and pentane were obtained through a solvent purification system. Anhydrous deuterated solvents were purchased from Eurisotop and stored over 4 Å molecular sieves. All chemicals, unless noted otherwise, were purchased from major commercial suppliers (TCI, Sigma-Aldrich) and used as received.

### NMR Spectrometry

NMR spectra were measured on a Bruker Avance II 400 MHz spectrometer. The following abbreviations are used for describing NMR spectra: s (singlet), d (doublet), t (triplet), td (triplet of doublets), ddd (doublet of doublets of doublets), vd (virtual doublet), vt (virtual triplet), br (broad). Chemical shifts (δ_H_, δ_C_) were quoted in parts per million (ppm) and were referenced to the residual solvent peak.

### Electrospray Ionization High-Resolution Mass Spectrometry (ESI-HRMS)

The samples were solubilized in methanol or MeCN and then injected in direct introduction (infusion) in the mass spectrometer. A Bruker mass spectrometer, model micrOTOF-Q II, was used with an electrospray source (ESI).

### Elemental Analyses

These were performed by José Manuel Pérez Falcón at the Microanalytical Facility at IIQ (Instituto de Investigaciones Químicas de Sevilla), using a LECO TruSpec CHN analyzer for determination of %C, %H and %N.

### Cyclic Voltammetry

he electrochemical experiments were performed under argon flow in a three-electrode cell. The working electrode (WE) was a steady glassy carbon electrode of approximately 0.07 cm^2^ surface area, the counter electrode (CE) was a platinum wire, and the reference electrode (WE) was a saturated calomel electrode separated from the solution by a fritz. The cyclic voltammograms (CVs) were recorded in dry CH_3_CN, using an AUTOLAB (Metrohm) PGSTAT100N or PGSTAT204 potentiostat run with Nova 2.1.4 software. The electrolyte salt, tetrabutylammonium hexafluorophosphate (TBAPF_6_) for electrochemical analysis, was purchased from Sigma-Aldrich and all the glassware was carefully dried before use_._

### Controlled Potential Electrolysis

Controlled potential electrolysis was conducted using an AUTOLAB PGSTAT302 (Metrohm). Preparative scale controlled potential electrolysis (CPE) experiments were performed in an electrolysis cell with a working compartment (4.5 mL liquid volume) and counter compartment (2 mL liquid volume) separated by an ultrafine glass frit, the total volume of the sealed cell is 25 mL, all CPEs were performed at +20 °C. A 2 cm^2^ glassy carbon plate was used as the working electrode, a platinum grid was used as the auxiliary electrode, and an Ag/Ag^+^ in a tipped glass tube filled with electrolyte (TBAPF_6_, 0.5 M in CH_3_CN) was used as a reference electrode. Both compartments were sealed to be gastight. A second glassy carbon electrode (0.03 cm^2^ area) was added to the working compartment to perform a CV scan before and after the CPE measurement. The working compartment was sparged with CO_2_ for 10 min before adding the solutions. The electrolyte solution was constantly stirred during the CPE experiment with a 1 cm stirring bar. No *iR* compensation was applied. The electrolysis experiments were then conducted at constant potential for the specified amount of time (3 h). After this period, the headspace of the cell was immediately analyzed by gas chromatography (GC).

### Photocatalytic CO2RR Experiments

These were performed in a quartz cuvette with a 1 cm width pathway (6 mL total vol. capacity) sealed with a rubber septum. All samples were prepared inside the glovebox in a 20 μM solution of the catalysts 1 to 4 in dry acetonitrile (3 mL), using BIH as ED [25 μM], Ru­(phen)_3_Cl_2_ [0.2 mM] as PS and TFE 10% V/V as H^+^D under Ar atmosphere and later bubbled with CO_2_ for 20 min to ensure total saturation. The photocatalytic reaction started by turning on the solar irradiation using an Oriel solar simulator model LSC-100 with 1 sun irradiation intensity, and a 420 nm optical filter was placed between the light source and the photochemical reactor. After 1 h of irradiation, samples of 110 μL were taken from the headspace (3 mL vol) using a gas thigh syringe and analyzed by GC, then left overnight to continue sampling the next day to complete 24 h of irradiation. Results of TON max after 24 h of irradiation are summarized in Table S9.

### Gas Detection

Gas Chromatography (GC) analysis of gas sampled from the headspace during the electrolysis was performed with an Agilent Technologies 7820A GC system equipped with a thermal conductivity detector. CO and H_2_ production was quantitatively detected using a CP-CarboPlot P7 capillary column (27.46 m in length and 25 μm internal diameter). Temperature was held at 150 °C for the detector and 34 °C for the oven. The carrier gas was argon flowing at 9.5 mL/min at a constant pressure of 0.4 bar. The injection was performed via a 250 μL gas-tight (Hamilton) syringe previously degassed with CO_2_. Conditions allowed the detection of both H_2_, O_2_, N_2_, CO, and CO_2_. Calibration curves for H_2_ and CO were determined separately by injecting known quantities of pure gas. Detection limits for CO and H_2_ are 5.2 × 10 ^–10^ and 1.6 × 10 ^–10^ mol, respectively.

### UV–Visible Spectro-Electrochemistry

UV–visible (UV–vis) spectroelectrochemical experiments were performed by monitoring the spectroscopic information obtained during Controlled Potential Electrolysis (CPE) experiments under argon. All the spectroscopic instrumentation was provided by Ocean Optics. UV–vis absorption spectra were recorded using a UV–vis probe (T300-RT-UV–vis) equipped with a dip-probe couple. The UV–vis probe was connected to an SR-6UUV400-50 spectrometer and a DH-2000 Deuterium–Halogen light source via optical fibers (600 nms). Spectroelectrochemical experiments were performed in a 4-electrode cell with a gas inlet with the UV–vis probe dipped in the solution under Ar and strong stirring. CPE experiments were performed using an AUTOLAB (Metrohm) PGSTAT100N potentiostat run with Nova 2.1.4 software. A glassy carbon plate was used as a working electrode (WE), platinum mesh as counter electrode (CE) and a Saturated Calomel Electrode (SCE) as reference electrode (RE). Both reference and counter electrode were separated from the solution with a fritz containing the electrolyte used on the experiment.

### X-ray Photoelectron Spectroscopy (XPS)

XPS experiments were performed in a PHOIBOS-100 spectrometer with a nonmonochromatic Mg- and a power source of 170 W. The electron energy hemispherical analyzer was operated in the constant pass energy mode (SPECS PHOIBOS 100DLD). Low resolution survey spectra were obtained with a pass energy = 50 eV, while high energy resolution spectra of detected elements were obtained with a pass energy = 30 eV. The spectra were analyzed with the CASA XPS software, version 2.3.16.Dev52 (Neal Fairly, UK).

### Ligand Synthesis

6-Methyl-2,2′:6′,2″-terpyridine carboxylate was synthesized following our previously reported procedure, starting from commercially available terpyridine.^32^ [2,2′:6′,2″-terpyridine]-6-carbaldehyde was synthesized following the procedures.^40^ [2,2′:6′,2″-terpyridine]-6-carbaldehyde (5.3 mmol, 1.4 g) and *trans*-1,2-cyclohexadiamine (2.66 mmol, 320 microL) were put inside a Schlenk under Are and dissolved in a mixture of dry MeOH/DCM (5 mL/10 mL). To this mixture, formic acid was added (20 microL) and was put to stir for 24 h at 55 °C. After 24 h, the solvent was removed under vacuum, and the solid was re-dissolved in a mixture of dry THF/MeOH (15 mL/10 mL). The solution was put in an ice bath, and NaBH_4_ (5 equiv) was slowly added due to a vigorous reaction. After all the NaBH_4_ was added, the Schlenk was placed in an oil bath, a reflux condenser was connected to the Schlenk and the reaction was left to stir at 50 °C overnight. After 24 h, the solvent was removed under vacuum, an aqueous saturated solution of NH_4_Cl (30 mL) was added and the compound was extracted in DCM (15 mL × 4). The combined organic layers were combined, dried (MgSO_4_) and the solvent was removed under vacuum after filtration. The ligand (1.5 g, 92%) was obtained as a light yellow solid and was used without further purification. ^1^H NMR (400 MHz, 20 °C, benzene-*d*_6_): 8.68 (m, 6H), 8.56 (m, 4H), 7.34 (m, 8H), 6.73 (m, 2H), 4.17 (d, ^2^ *J*_H,H_ = 15 Hz, 2H), 3.99 (d, ^2^ *J*_H,H_ = 15 Hz, 2H), 2.51 (m, 2H), 2.09 (m, 2H), 1.54 (br, 2H) and 1.08 (m, 4H) ppm. The signals at 1.2 and 0.8 ppm correspond to pentane. ^13^C­{^1^H} NMR (101 MHz, 20 °C, benzene-*d*_6_): 159.8, 156.4, 155.6, 155.5, 155.5, 149.0, 137.5, 136.8, 136.1, 123.3, 122.2, 121.1, 120.9, 120.8, 119.0, 60.9, 51.9, 31.2, 24.9 ppm. ESI-HRMS (*m*/*z* pos): Found (Calcd): C_38_H_37_N_8_^+^ 605.3124 (605.3136).

### Complex **1**

Ligand **L** (100 mg, 0.165 mmol) was dissolved in 3 mL of dry THF inside the glovebox, and a solution of the metal precursor [Fe­(MeCN)_4_]­[BF_4_]_2_ (60 mg, 0.150 mmol) in 3 mL of dry MeCN was slowly added. The strong violet solution was left to stir for 2 h at room temperature. After this time, the solvent was removed under vacuum inside the glovebox, and the solid was thoroughly washed with toluene (5 × 3 mL). The remaining purple solid was vacuum-dried, generating complex **1** in 97% yield (120 mg). This solid provided a positive elemental analysis. Elemental Analysis. Found: 54.79 C, 4.48 H, 13.43N. Theoretical for [Fe­(L)]­[BF_4_]_2_: 54.71 C, 4.35 H, 13.43 N. ESI-HRMS (*m*/*z* pos): Found (Calcd): C_38_H_36_N_8_Fe^2+^ 330.1195 (330.1201).

### Complex **2**

Ligand **L** (100 mg, 0.165 mmol) was dissolved in 3 mL of dry THF inside the glovebox, and a solution of the metal precursor [Ni­(MeCN)_4_]­[BF_4_]_2_ (60 mg, 0.150 mmol) in 3 mL of dry MeCN was slowly added. The yellow solution was left to stir for 2 h at room temperature. After this time, the solvent was removed under vacuum inside the glovebox, and the solid was thoroughly washed with toluene (5 × 3 mL). The remaining yellow solid was vacuum-dried, generating complex **2** in 95% yield (118 mg). This solid provided a positive elemental analysis. Elemental Analysis. Found: 54.62 C, 4.41 H, 13.09N. Theoretical for [Ni­(L)]­[BF_4_]_2_: 54.53 C, 4.34 H, 13.39 N. ESI-HRMS (*m*/*z* pos): Found (Calcd): C_38_H_36_N_8_Ni^2+^ 331.1200 (331.1203).

### Complex **3**

Complex **1** (100 mg, 0.119 mmol) was dissolved in 3 mL of dry MeCN inside the glovebox, and a solution of the metal precursor [Cu­(MeCN)_4_]­[BF_4_] (39 mg, 0.122 mmol) in 3 mL of dry MeCN was slowly added. The purple solution became dark red instantaneously, and it was left to stir for 2 h at room temperature. After this time, the solvent was removed under vacuum inside the glovebox, and the solid was thoroughly washed with THF (5 × 3 mL). The remaining brown–reddish solid was dissolved in a mixture of MeCN/Toluene, filtered and left to slowly evaporate, obtaining large red crystals of complex **3** in 80% yield (102 mg). This solid provided a positive elemental analysis. Elemental Analysis. Found: 47.28 C, 3.92 H, 13.31 N. Theoretical for [FeCu­(L)­(MeCN)_2_]­[BF_4_]_3_: 47.29 C, 3.97 H, 13.13 N. ESI-HRMS (*m*/*z* pos): Found (Calcd): C_38_H_36_FeCuN_8_^3+^ Not found.

### Complex **4**

Complex **2** (100 mg, 0.119 mmol) was dissolved in 3 mL of dry MeCN inside the glovebox, and a solution of the metal precursor [Cu­(MeCN)_4_]­[BF_4_] (39 mg, 0.122 mmol) in 3 mL of dry MeCN was slowly added. The yellow solution became dark orange instantaneously, and it was left to stir for 2 h at room temperature. After this time, the solvent was removed under vacuum inside the glovebox, and the solid was thoroughly washed with THF (5 × 3 mL). The remaining brown solid was dissolved in a mixture of MeCN/Toluene, filtered and left to slowly evaporate, obtaining dark orange crystals of complex **4** in 78% yield (99 mg). This solid provided a positive elemental analysis. Elemental Analysis. Found: 47.47 C, 3.71 H, 12.72 N. Theoretical for [NiCu­(L)­(MeCN)_2_]­[BF_4_]_3_: 47.17 C, 3.96 H, 13.10 N. ESI-HRMS (*m*/*z* pos): Found (Calcd): C_38_H_36_NiCuN_8_^3+^ Not found.

## Supplementary Material



## Notes

Experimental spectra and crystallographic data (PDF)
