# Supplementary material for: Access to Heterobimetallic MII/CuI Complexes with a Multichelate Platform and Their Reactivity Studies in CO2RR
Source: Inorg Chem. 2025 Mar 3;64(10):4835–43. doi: 10.1021/acs.inorgchem.4c04471 (PMC12135035; doi:10.1021/acs.inorgchem.4c04471)
Supplement: Supplementary file 1 [file ic4c04471_si_001.pdf]

Supporting Information

Access to heterobimetallic M^{II}/Cu^{I} complexes with a multichelate platform and their reactivity studies in CO2RR

Samantha L Peralta–Arriaga,[‡,□] *Miguel Ángel Martín–Neri* [‡,□] *Carlos García Bellido,*[□]
Jeremy De Freitas,[γ] *Sukanta Saha,*[γ] *Francisco José Fernández–de–Córdova,*[□] *Marc Robert,* [□,ξ,γ] *and Orestes Rivada–Wheelaghan* [□,□] *.

[□] Université Paris Cité, Laboratoire d'Electrochimie Moléculaire, CNRS, F–75006 Paris, France.

[γ] Sorbonne Université, CNRS, Institut Parisien de Chimie Moléculaire, F–75005, Paris, France.

[□] Instituto de Investigaciones Químicas (IIQ), Departamento de química Inorgánica, Universidad de Sevilla, Avenida Américo Vespucio 49, 41092, Sevilla, Spain.

* Corresponding author : orivada@us.es

[ξ] Institut Universitaire de France (IUF), F–75005 Paris, France.

Table of contents

1. Experimental Details
    a. X–Ray crystallography……………………………………………………S3
2. Characterization of ligand, **L**, and complexes
    a. Ligand, **L**……………………………………………………………………S5
    b. Complex **1**, [FeL][BF_4]$_2$………………………………………………..S12
    c. Complex **2**, [NiL][BF_4]$_2$…………………………………………….…S8
    d. Complex **3**, [FeCuL(MeCN)$_2$][BF_4]$_3$………………………………..…S16
    e. Complex **4**, [NiCuL(MeCN)$_2$][BF_4]$_3$………………………………..…S19
3. Cyclic voltammetry…………………………………………………………..S22
4. UV–visible spectro−electrochemical experiments and comparison with chemical reduction………………………………………………………..…S33
5. Calculation of number of electrons…………………………………….…S35
6. Controlled potential electrolysis experiments…………………………………S36
7. Photocatalytic CO2RR experiments……………………………………………S51

X−Ray crystallography

The data for **2**: Low–temperature diffraction data were collected on a Bruker D8 Quest APEX–III single crystal diffractometer with a Photon III detector and a IμS 3.0 microfocus X–ray source at the Instituto de Investigaciones Químicas, Sevilla. Data were collected by means of ω and φ scans using monochromatic radiation λ(Mo Kα1) = 0.71073 Å. The diffraction images collected were processed and scaled using APEX4 v2021.10–0 software. The structures were solved with SHELXT and was refined against F2 on all data by full–matrix least squares with SHELXL [1], using Olex2 [2] as graphical interface. All non–hydrogen atoms were refined anisotropically. Hydrogen atoms were included in the model at geometrically calculated positions and refined using a riding model, unless otherwise noted. The isotropic displacement parameters of all hydrogen atoms were fixed to 1.2 times the U value of the atoms to which they are linked (1.5 times for methyl groups). The hydrogen of amine groups were located from difference Fourier maps and included in a refinement with isotropic parameters

The data for **3:** Low–temperature diffraction data were collected on a Bruker D8 Quest APEX–III single crystal diffractometer with a Photon III detector and a IμS 3.0 microfocus X–ray source at the Instituto de Investigaciones Químicas, Sevilla. Data were collected by means of ω and φ scans using monochromatic radiation λ(Mo Kα1) = 0.71073 Å. The diffraction images collected were processed and scaled using APEX–4 v2021.4–0 software. The structures were solved with SHELXT and was refined against F2 on all data by full–matrix least squares with SHELXL [1], using Olex2 [2] as graphical interface. All non–hydrogen atoms were refined anisotropically. Hydrogen atoms were included in the model at geometrically calculated positions and refined using a riding model, unless otherwise noted. The isotropic displacement parameters of all hydrogen atoms were fixed to 1.2 times the U value of the atoms to which they are linked (1.5 times for methyl groups).

The data for **4**: Low–temperature diffraction data were collected on a Bruker D8 Quest APEX–III single crystal diffractometer with a Photon III detector and a IμS 3.0 microfocus X–ray source at the Instituto de Investigaciones Químicas, Sevilla. Data were collected by means of ω and φ scans using monochromatic radiation λ(Mo Kα1) = 0.71073 Å. The diffraction images collected were processed and scaled using APEX–4 v2021.4–0 software. The structures were solved with SHELXT and was refined against F2 on all data by full–matrix least squares with SHELXL [1], using Olex2 [2] as graphical interface. All non–hydrogen atoms were refined anisotropically. Hydrogen atoms were included in the model at geometrically calculated positions and refined using a riding model, unless otherwise noted. The isotropic displacement parameters of all hydrogen atoms were fixed to 1.2 times the U value of the atoms to which they are linked (1.5 times for methyl groups).

[1] Sheldrick, G. M. Crystal structure refinement with SHELXL. *Acta Cryst.* **2008**, *A64*, 112–122.

[2] Dolomanov, O.V., Bourhis, L.J., Gildea, R.J, Howard, J.A.K. & Puschmann, H. *J. Appl. Cryst*. **2009**, *42*, 339–341.

Ligand, L

N[1],N[2]–bis([2,2':6',2''–terpyridin]–6–ylmethyl)cyclohexane–1,2–diamine

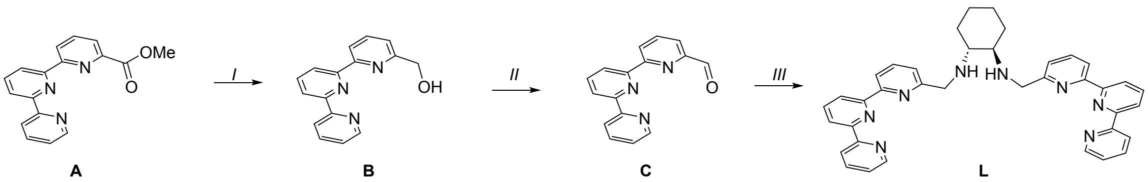

Scheme S1. Synthesis of ligand **L**. *I*, carboxylate reduction (**B**);[1] *II*, alcohol oxidation (**C**);[1] *III*, ligand formation (**L**).[2]

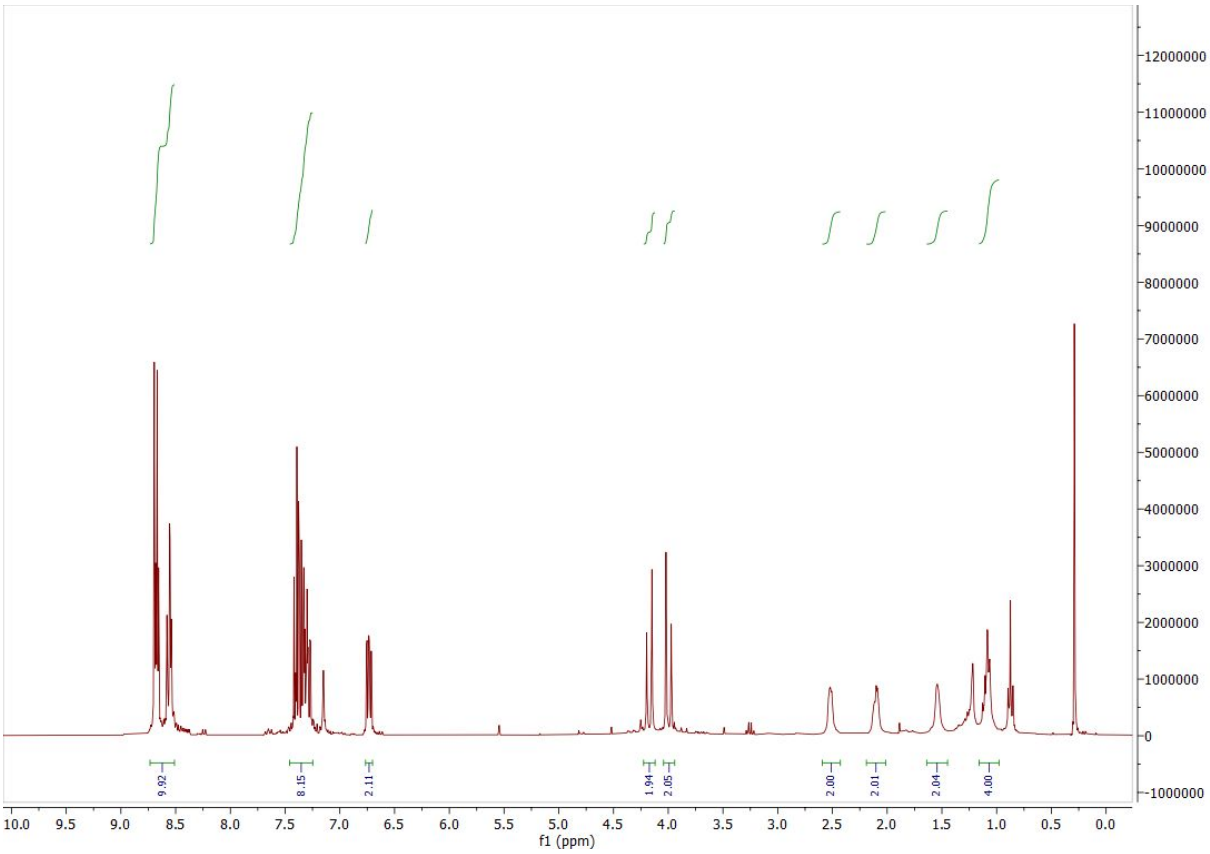

Figure S1. ^{1}H NMR spectrum (400 MHz) of ligand **L** in benzene–d$_6$ solution. Signals for pentane are observed at 0.8 and 1.2 ppm aprox.

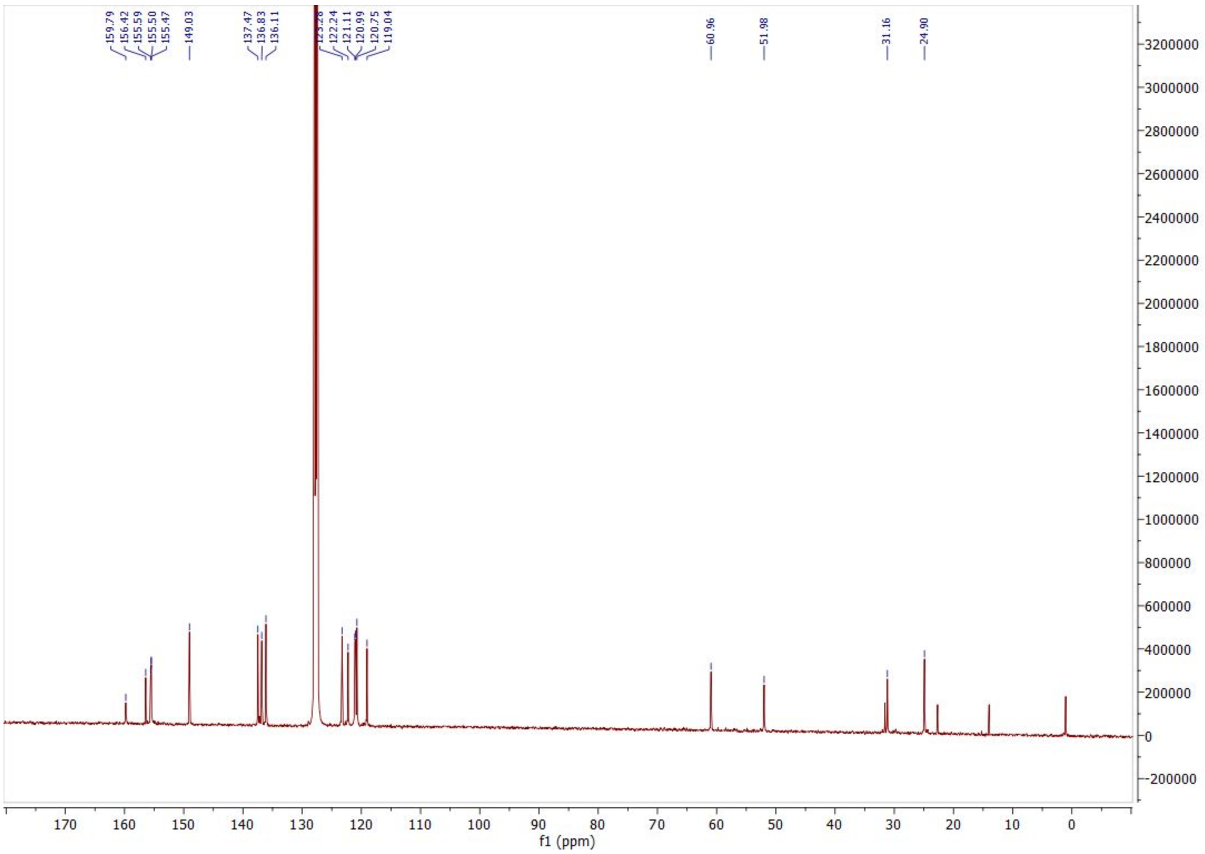

Figure S2. $^{13}C\{^{1}H\}$ spectrum (101 MHz) of **L** in benzene–d_6 solution. Signals for pentane are observed at 31.6, 22.7 and 13.9 ppm.

UV–vis spectroscopy

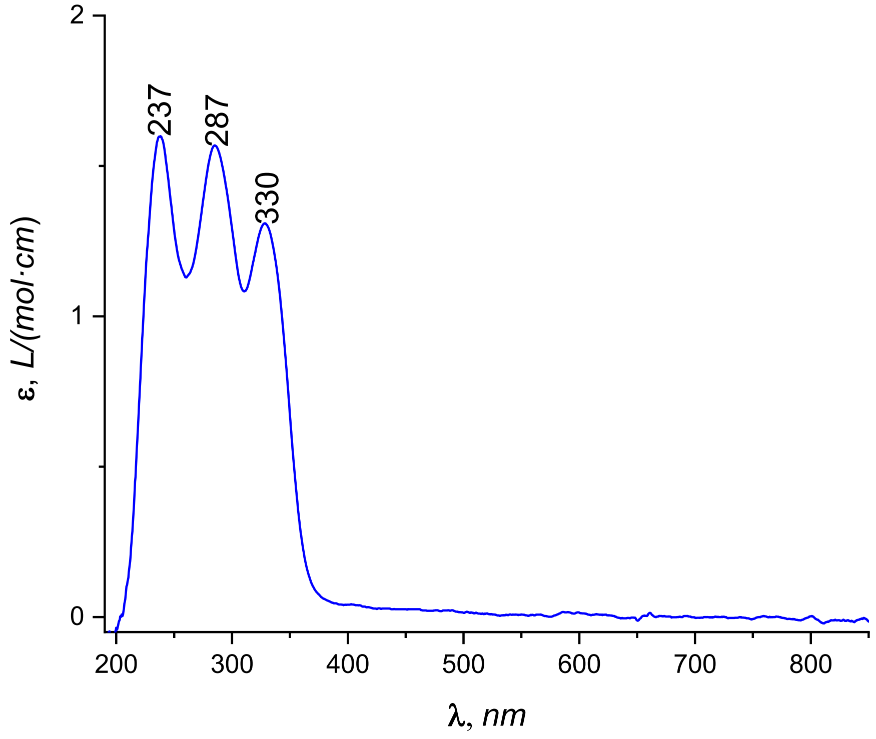

Figure S3. UV–visible spectra of ligand **L** in THF, the thickness of the quartz cell is 1 cm (40 μM).

Experimental Mass Spectrum

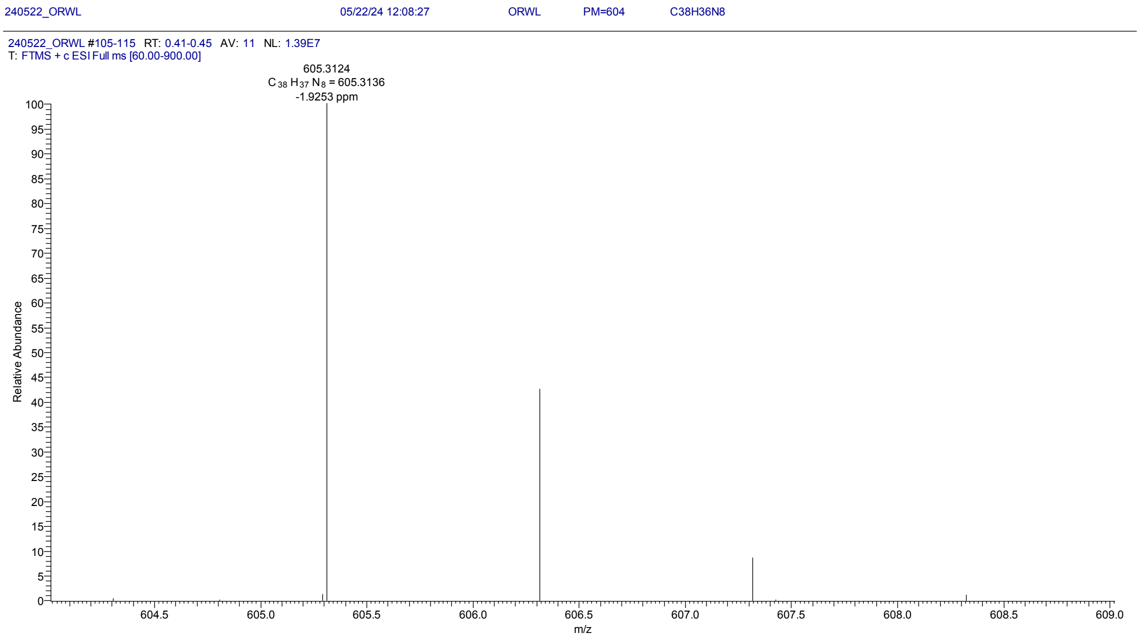

Figure S4. Experimental ESI–MS spectrum of the ligand **L**.

Complex 1, [FeL][BF$_4$]$_2$

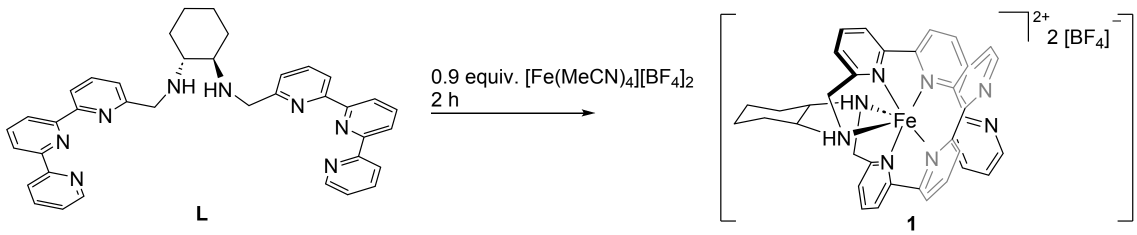

Scheme S2. Synthesis of complex **1**.

Experimental Mass Spectrum

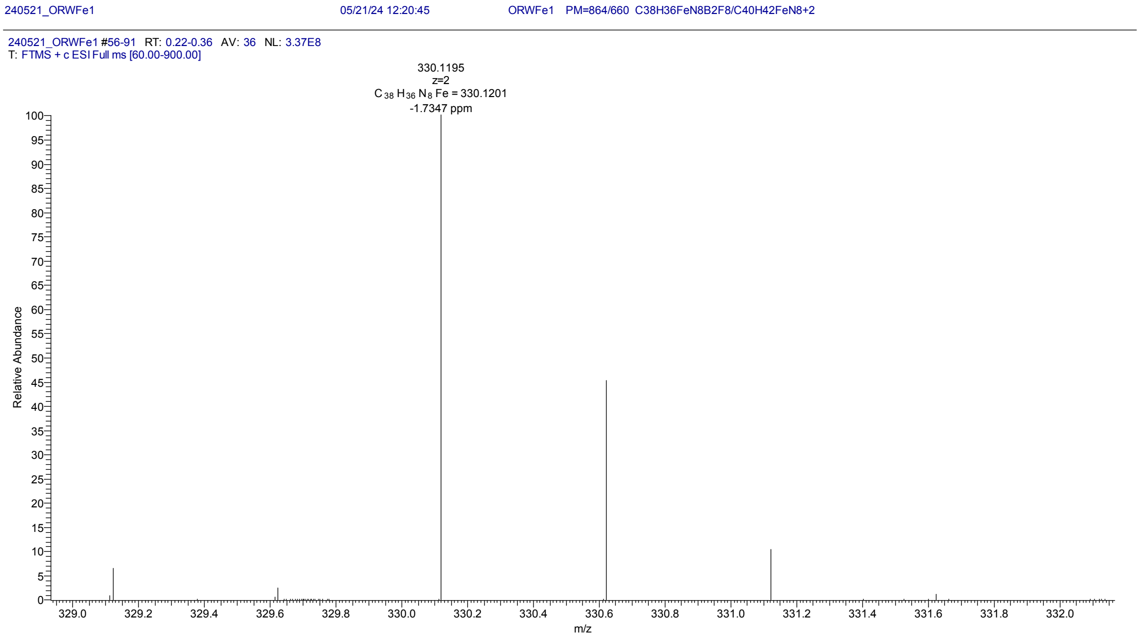

Figure S5. Experimental ESI–MS spectrum of **1**.

UV–vis spectroscopy

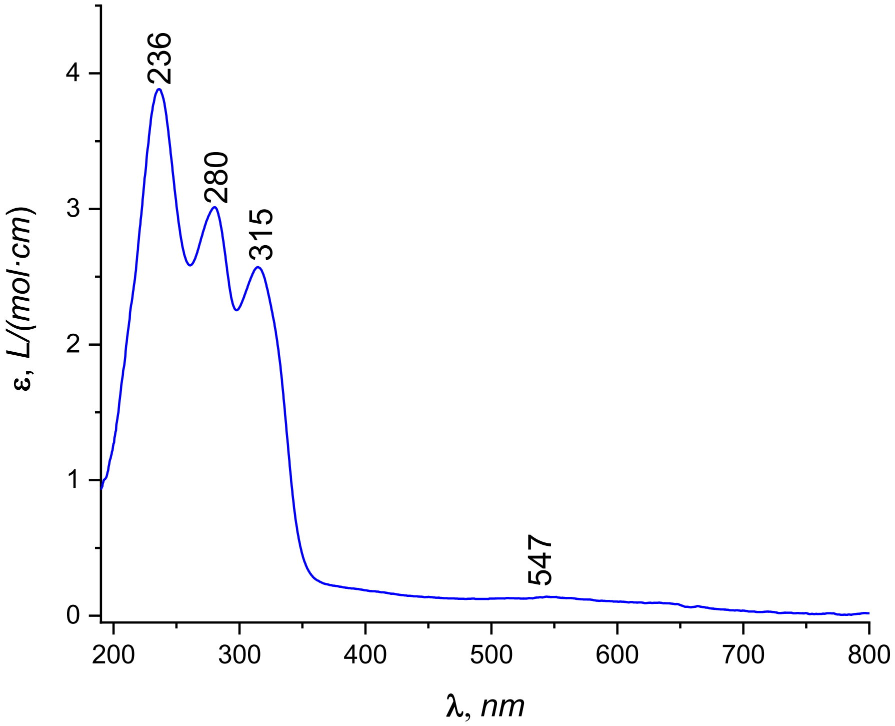

Figure S6. UV–visible spectra of complex **1** in CH_3CN, the thickness of the quartz cell is 1 cm (40 μM).

1. Measurement of magnetic moment by the Evans method

Equation (1):

$$\chi_g = \chi_0 \cdot \frac{3000 \cdot \Delta\upsilon}{4 \cdot \pi \cdot \upsilon_0 \cdot c \cdot M} + \frac{\chi_0 \cdot (\rho_0 - \rho_s)}{c} \qquad (1)$$

Where:

χ_g = mass susceptibility of the solute

χ_0 = mass susceptibility of the solvent

$\Delta\upsilon$ = observed frequency shift of the reference resonance (Hz)

υ_0 = spectrometer frequency (Hz)

c = concentration of the substance in mol/L

ρ_0 = density of the pure solvent

ρ_s = density of the solution

M = molecular weight

Assuming that $\rho_0 - \rho_s$ is zero and then multiplying by M simplifies to give Equation (2):

$$\chi_M = \frac{3000 \cdot \Delta \upsilon}{4 \cdot \pi \cdot \upsilon_0 \cdot c} \qquad (2)$$

Where χ_M = molar susceptibility of the solute ($cm^3 \cdot mol^{-1}$).

The uncorrected effective magnetic moment, μ_{eff}, has been calculated from the molar susceptibility using equation (3) without considering the diamagnetic contributions:

$$\mu_{eff} = 2.828 \cdot \sqrt{\chi_M \cdot T} \qquad (3)$$

Mass of **1** = 5.4 mg

M = 864.29 g/mol

Solvent = 0.35 mL of CD_3CN solution containing mesitylene as internal standards.

$\Delta \upsilon$ = 195 Hz for both signals of mesitylene.

υ_0 = 300 MHz

c = 0.018 mol/L

T = 298 K

Using mesitylene signal shifts:

χ_M = 0.009 $cm^3 \cdot mol^{-1}$

μ_{eff} = 4.55 (BM)

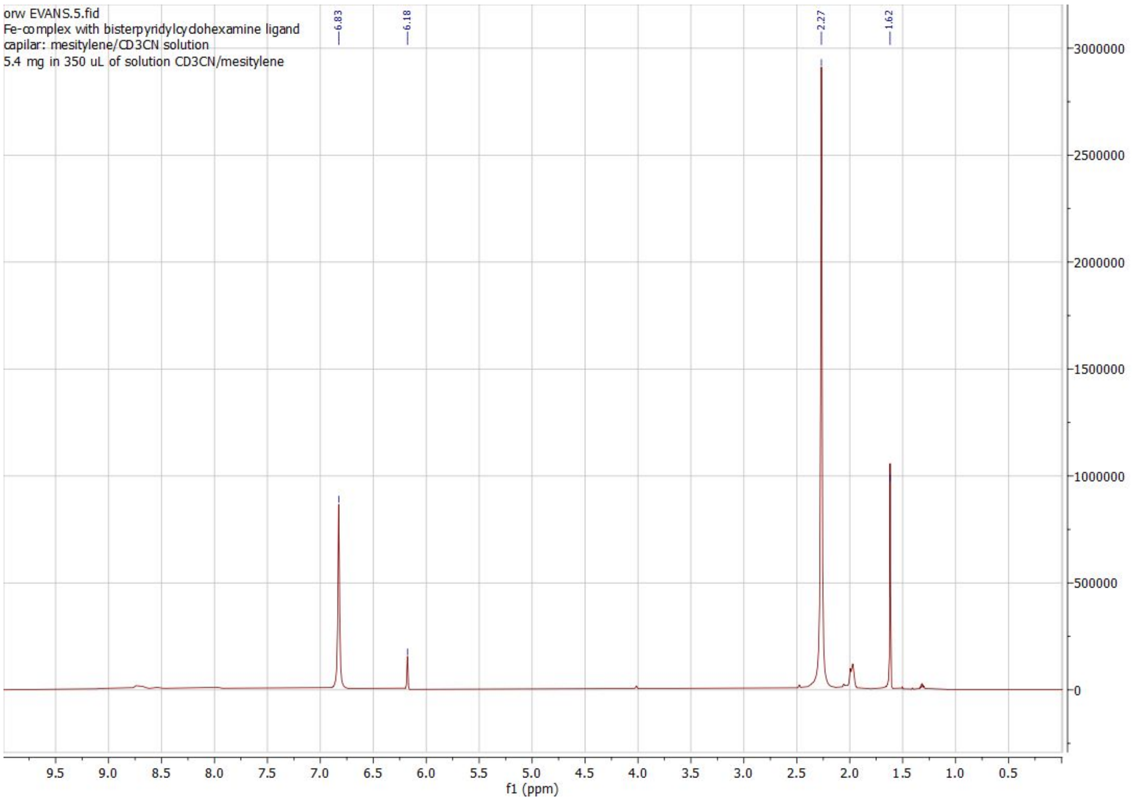

Figure S7. ^{1}H NMR spectrum (400 MHz) of a CD_3CN solution containing mesitylene (6.83/5.97 and 2.27/1.41 ppm) as internal standards and complex **1** (19 mM).

Solutions of 1mM in MeCN of **1** were silent when analysed by electron paramagnetic resonance at liquid N_2 temperature.

Complex 2, [NiL][BF$_4$]$_2$

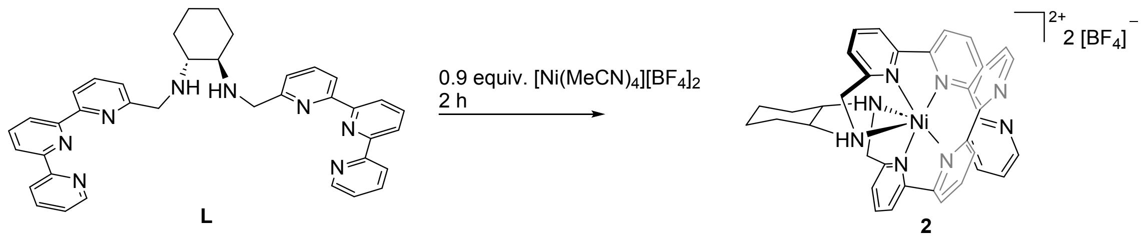

Scheme S3. Synthesis of complex **2**.

Experimental Mass Spectrum

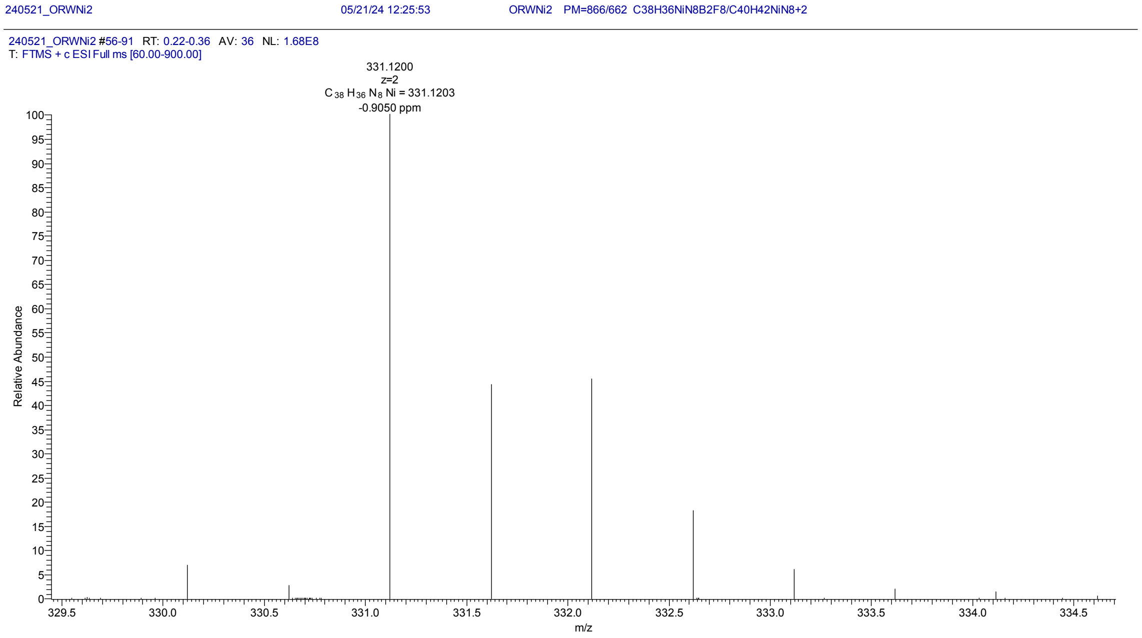

Figure S8. Experimental ESI–MS spectrum of **2**.

UV–vis spectroscopy

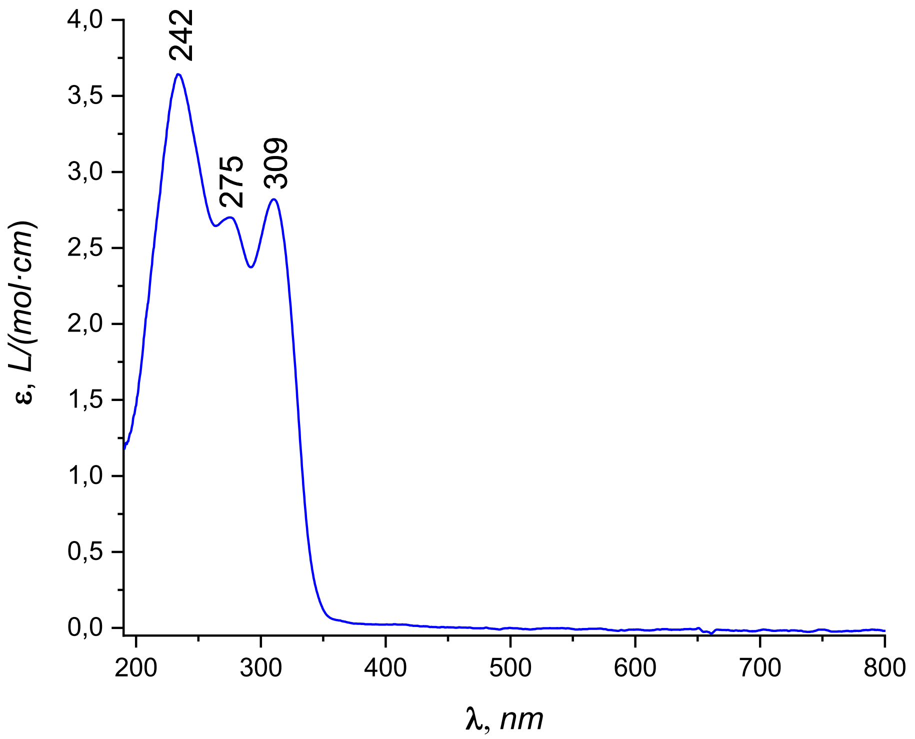

Figure S9. UV–visible spectra of complex **2** in CH_3CN, the thickness of the quartz cell is 1 cm (40 μM).

2. Measurement of magnetic moment by the Evans method

Equation (1):

$$\chi_g = \chi_0 \cdot \frac{3000 \cdot \Delta\upsilon}{4 \cdot \pi \cdot \upsilon_0 \cdot c \cdot M} + \frac{\chi_0 \cdot (\rho_0 - \rho_s)}{c} \qquad (1)$$

Where:

χ_g = mass susceptibility of the solute

χ_0 = mass susceptibility of the solvent

$\Delta\upsilon$ = observed frequency shift of the reference resonance (Hz)

υ_0 = spectrometer frequency (Hz)

c = concentration of the substance in mol/L

ρ_0 = density of the pure solvent

ρ_s = density of the solution

M = molecular weight

Assuming that $\rho_0 - \rho_s$ is zero and then multiplying by M simplifies to give Equation (2):

$$\chi_M = \frac{3000 \cdot \Delta \upsilon}{4 \cdot \pi \cdot \upsilon_0 \cdot c} \qquad (2)$$

Where χ_M = molar susceptibility of the solute ($cm^3 \cdot mol^{-1}$).

The uncorrected effective magnetic moment, μ_{eff}, has been calculated from the molar susceptibility using equation (3) without considering the diamagnetic contributions:

$$\mu_{eff} = 2.828 \cdot \sqrt{\chi_M \cdot T} \qquad (3)$$

Mass of **2** = 4.4 mg

M = 866.29 g/mol

Solvent = 0.35 mL of CD_3CN solution containing mesitylene as internal standards.

$\Delta \upsilon$ = 53 Hz for both signals of mesitylene.

υ_0 = 300 MHz

c = 0.015 mol/L

T = 298 K

Using mesitylene signal shifts:

χ_M = 0.003 $cm^3 \cdot mol^{-1}$

μ_{eff} = 2.67 (BM)

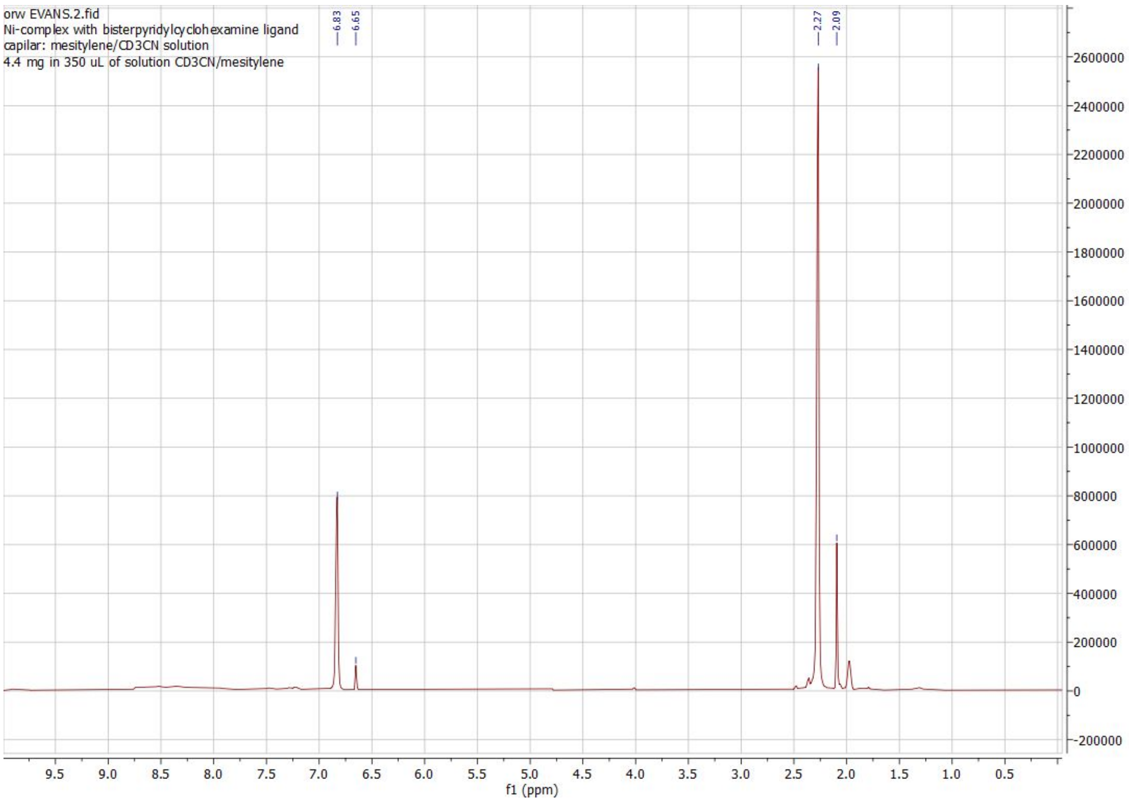

Figure S10. ^{1}H NMR spectrum (400 MHz) of a CD_3CN solution containing mesitylene (6.83/6.65 and 2.27/2.09 ppm) as internal standards and complex **1** (15 mM).

Solutions of 1mM in MeCN of **2** were silent when analysed by electron paramagnetic resonance at liquid N_2 temperature.

Complex 3, [FeCuL(MeCN)$_2$][BF$_4$]$_3$

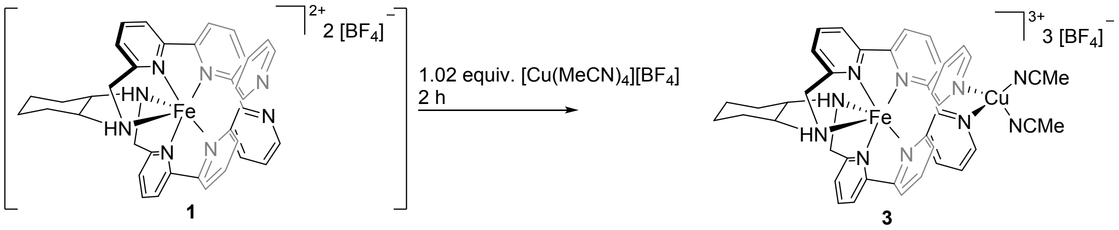

Scheme S4. Synthesis of complex **3**.

UV–vis spectroscopy

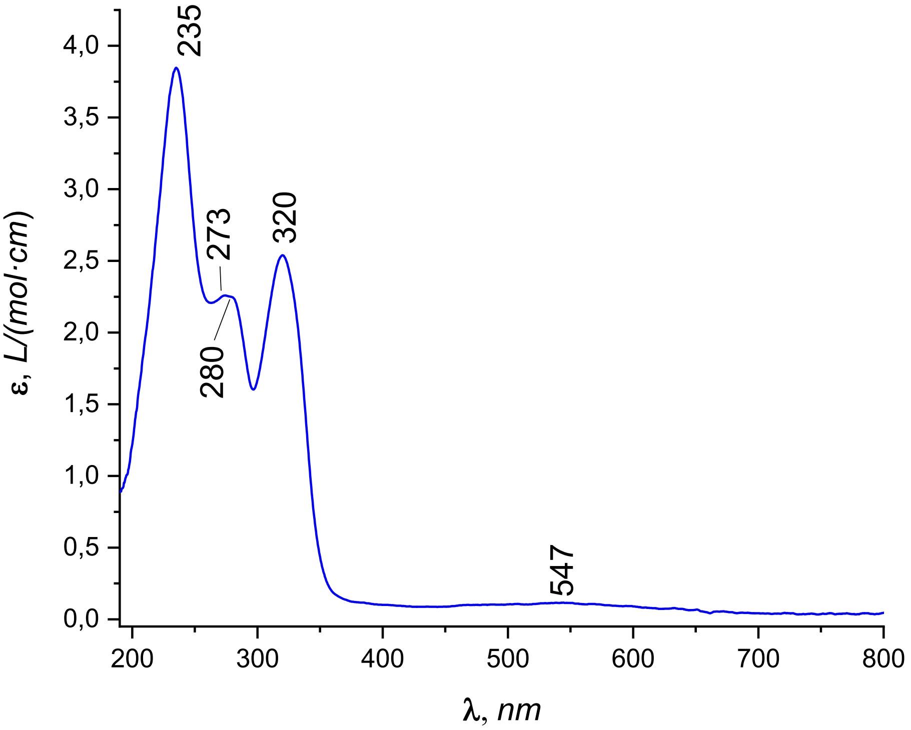

Figure S11. UV–visible spectra of complex **3** in CH_3CN, the thickness of the quartz cell is 1 cm (40 μM).

3. Measurement of magnetic moment by the Evans method

Equation (1):

$$\chi_g = \chi_0 \cdot \frac{3000 \cdot \Delta\upsilon}{4 \cdot \pi \cdot \upsilon_0 \cdot c \cdot M} + \frac{\chi_0 \cdot (\rho_0 - \rho_s)}{c} \qquad (1)$$

Where:

χ_g = mass susceptibility of the solute

χ_0 = mass susceptibility of the solvent

$\Delta\upsilon$ = observed frequency shift of the reference resonance (Hz)

υ_0 = spectrometer frequency (Hz)

c = concentration of the substance in mol/L

ρ_0 = density of the pure solvent

ρ_s = density of the solution

M = molecular weight

Assuming that $\rho_0 - \rho_s$ is zero and then multiplying by M simplifies to give Equation (2):

$$\chi_M = \frac{3000 \cdot \Delta\upsilon}{4 \cdot \pi \cdot \upsilon_0 \cdot c} \qquad (2)$$

Where χ_M = molar susceptibility of the solute ($cm^3 \cdot mol^{-1}$).

The uncorrected effective magnetic moment, μ_{eff}, has been calculated from the molar susceptibility using equation (3) without considering the diamagnetic contributions:

$$\mu_{eff} = 2.828 \cdot \sqrt{\chi_M \cdot T} \qquad (3)$$

Mass of **3** = 5.6 mg

M = 1096.28 g/mol

Solvent = 0.35 mL of CD_3CN solution containing mesitylene as internal standards.

$\Delta\upsilon$ = 151 Hz for both signals of mesitylene.

υ_0 = 300 MHz

c = 0.015 mol/L

T = 298 K

Using mesitylene signal shifts:

χ_M = 0.008 $cm^3 \cdot mol^{-1}$

μ_{eff} = 4.43 (BM)

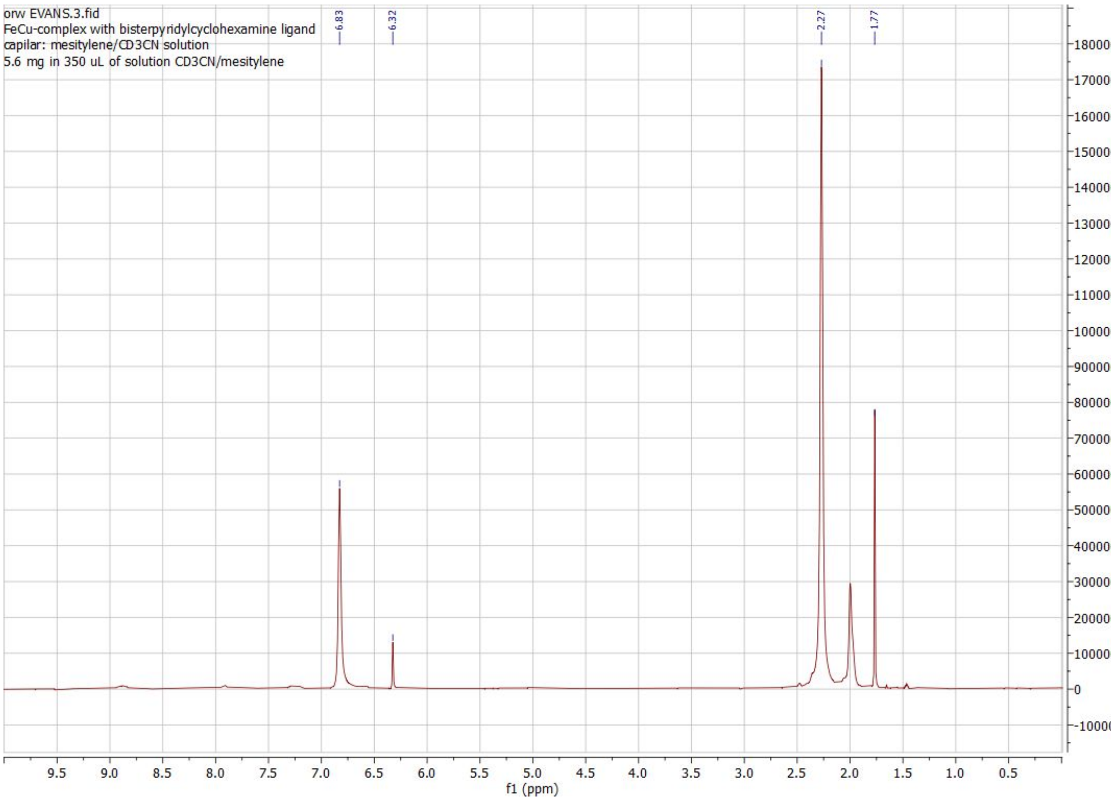

Figure S12. ^{1}H NMR spectrum (400 MHz) of a CD_3CN solution containing mesitylene (6.83/6.32 and 2.27/1.77 ppm) as internal standards and complex **3** (15 mM).

Solutions of 1mM in MeCN of **3** were silent when analysed by electron paramagnetic resonance at liquid N_2 temperature.

Complex 4, $[NiCuL(MeCN)_2][BF_4]_3$

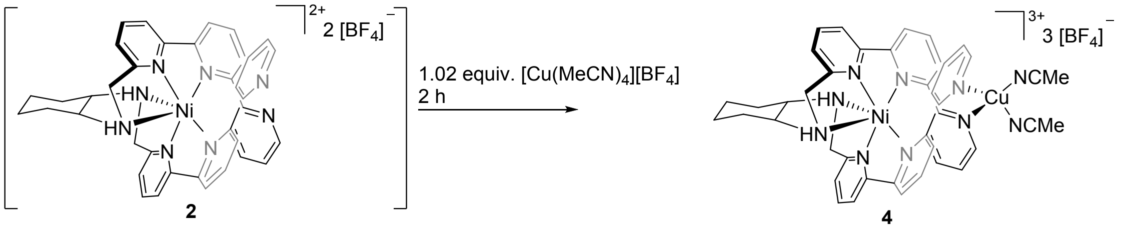

Scheme S5. Synthesis of complex **3**.

UV–vis spectroscopy

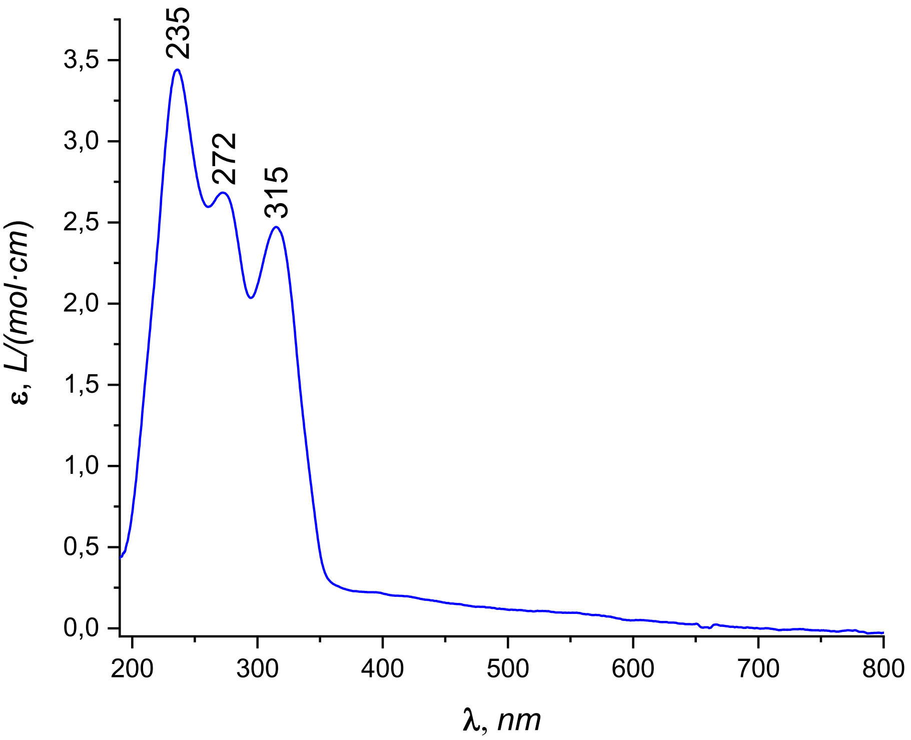

Figure S13. UV–visible spectra of complex **4** in CH_3CN, the thickness of the quartz cell is 1 cm (40 μM).

4. Measurement of magnetic moment by the Evans method

Equation (1):

$$\chi_g = \chi_0 \cdot \frac{3000 \cdot \Delta\upsilon}{4 \cdot \pi \cdot \upsilon_0 \cdot c \cdot M} + \frac{\chi_0 \cdot (\rho_0 - \rho_s)}{c} \qquad (1)$$

Where:

χ_g = mass susceptibility of the solute

χ_0 = mass susceptibility of the solvent

$\Delta\upsilon$ = observed frequency shift of the reference resonance (Hz)

υ_0 = spectrometer frequency (Hz)

c = concentration of the substance in mol/L

ρ_0 = density of the pure solvent

ρ_s = density of the solution

M = molecular weight

Assuming that $\rho_0 - \rho_s$ is zero and then multiplying by M simplifies to give Equation (2):

$$\chi_M = \frac{3000 \cdot \Delta\upsilon}{4 \cdot \pi \cdot \upsilon_0 \cdot c} \qquad (2)$$

Where χ_M = molar susceptibility of the solute ($cm^3 \cdot mol^{-1}$).

The uncorrected effective magnetic moment, μ_{eff}, has been calculated from the molar susceptibility using equation (3) without considering the diamagnetic contributions:

$$\mu_{eff} = 2.828 \cdot \sqrt{\chi_M \cdot T} \qquad (3)$$

Mass of **4** = 6.9 mg

M = 1098.29 g/mol

Solvent = 0.35 mL of CD_3CN solution containing mesitylene as internal standards.

$\Delta\upsilon$ = 75 Hz for both signals of mesitylene.

υ_0 = 300 MHz

c = 0.018 mol/L

T = 298 K

Using mesitylene signal shifts:

χ_M = 0.003 $cm^3 \cdot mol^{-1}$

μ_{eff} = 2.67 (BM)

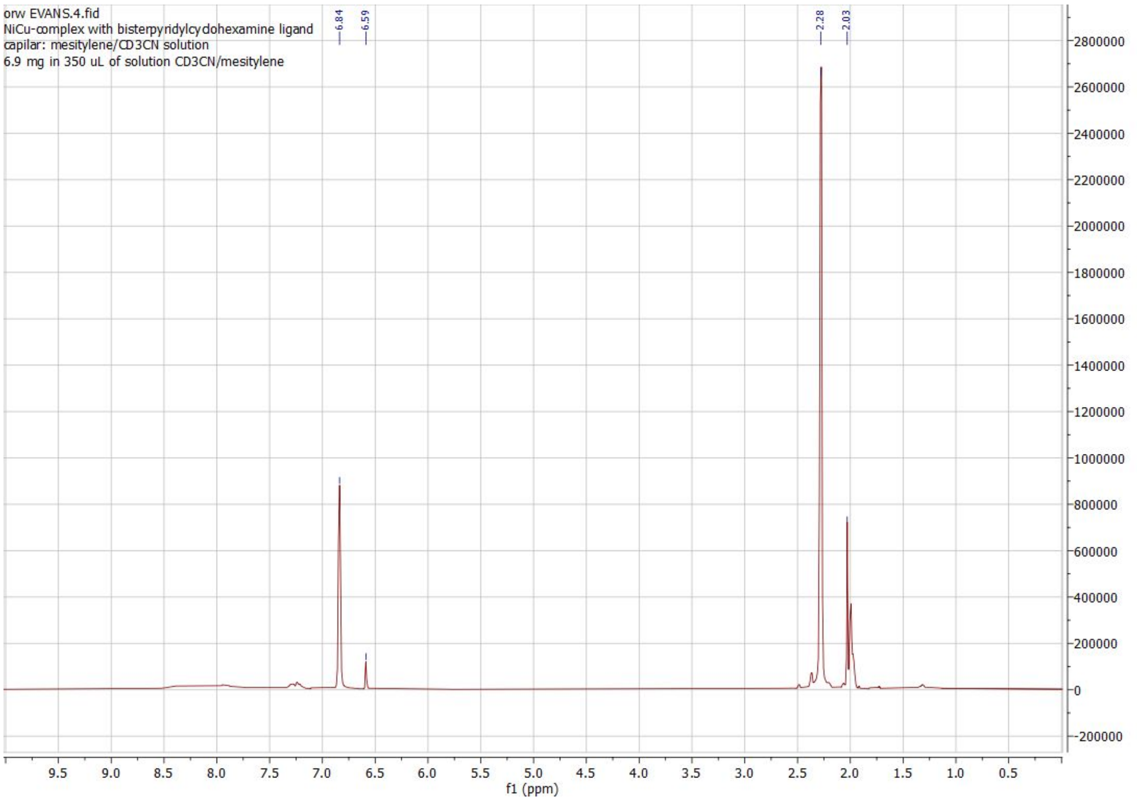

Figure S14. ^{1}H NMR spectrum (400 MHz) of a CD_3CN solution containing mesitylene (6.84/6.59 and 2.28/2.03 ppm) as internal standards and complex **4** (18 mM).

Solutions of 1mM in MeCN of **4** were silent when analysed by electron paramagnetic resonance at liquid N_2 temperature.

Cyclic voltammetry

All cyclic voltammetry measurements were performed under the same conditions using dry acetonitrile and $[NBu_4][PF_6]$ 0.1M as support electrolyte solution in a conventional three–electrode cell with a Saturated Calomel Electrode (SCE) as reference electrode (RE) (at constant T = 293 K), Pt wire as counter electrode (CE) and glassy carbon as working electrode (WE). All the measurements were performed at 0.1 $V{\cdot}s^{-1}$ unless stated otherwise.

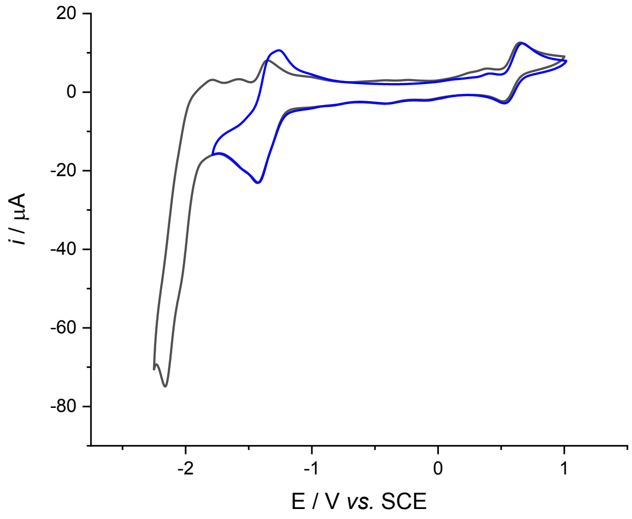

Figure S15. Cyclic voltammetry under Argon of **1** in anhydrous acetonitrile with 0.1 M of $TBAPF_6$ at 25°C at 0.1 $V{\cdot}s^{-1}$, [**1**] = 1 mM.

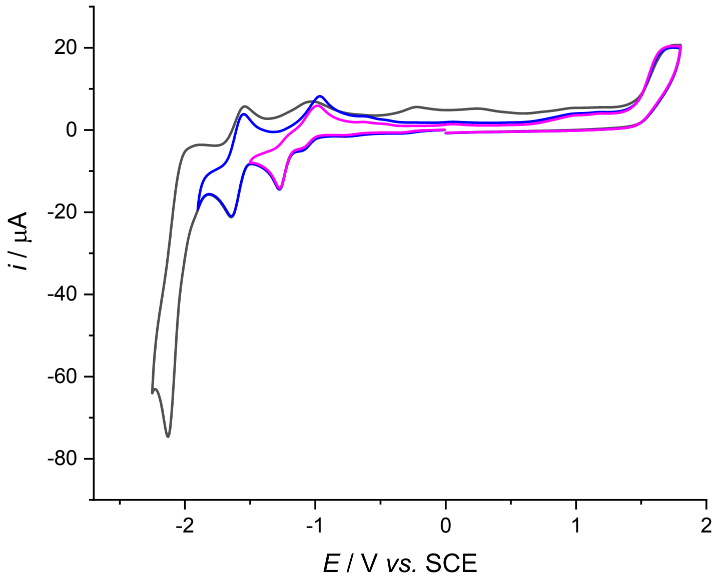

Figure S16. Cyclic voltammetry under Argon of **2** in anhydrous acetonitrile with 0.1 M of $TBAPF_6$ at 25°C at 0.1 $V{\cdot}s^{-1}$, [**1**] = 1 mM.

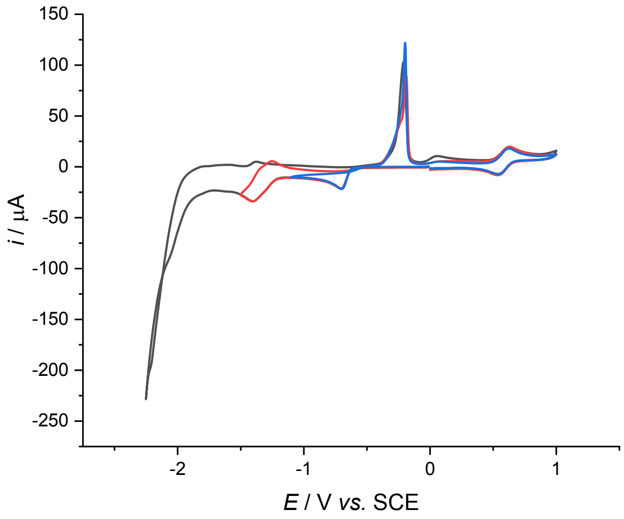

Figure S17. Cyclic voltammetry under Argon of **3** in anhydrous acetonitrile with 0.1 M of $TBAPF_6$ at 25°C at 0.1 $V{\cdot}s^{-1}$, [**1**] = 1 mM.

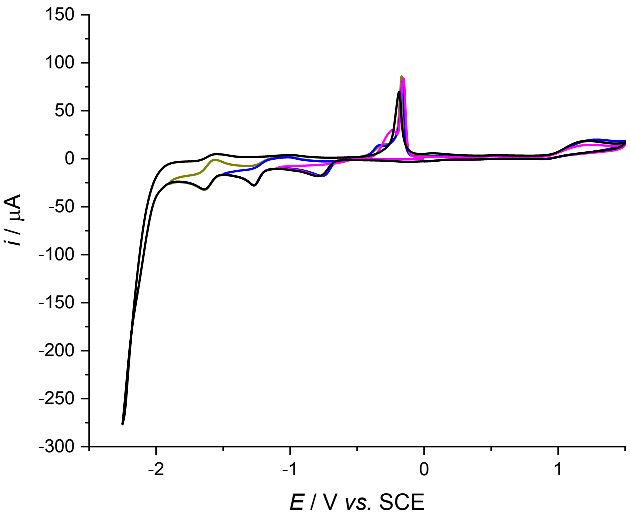

Figure S18. Cyclic voltammetry under Argon of **4** in anhydrous acetonitrile with 0.1 M of $TBAPF_6$ at 25°C at 0.1 $V{\cdot}s^{-1}$, [**1**] = 1 mM.

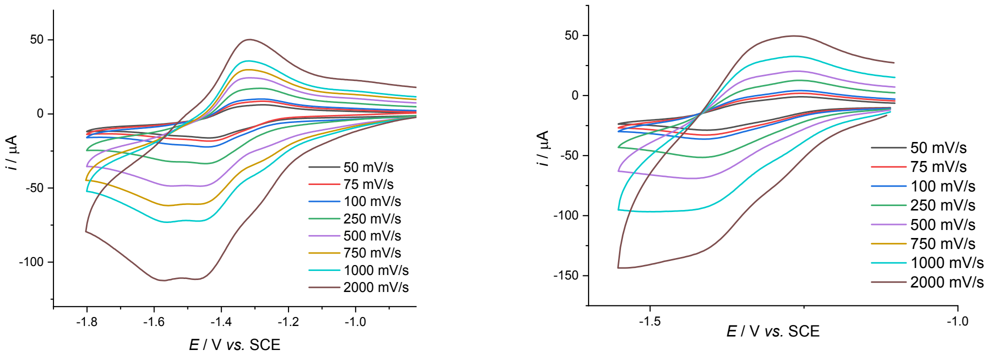

Figure S19. Scan rate studies from 0.05 to 2 V·s^{-1} of 1st reduction of complexes **1** (left) and **3** (right) in anhydrous acetonitrile with 0.1 M of $TBAPF_6$ at 25°C under Ar,

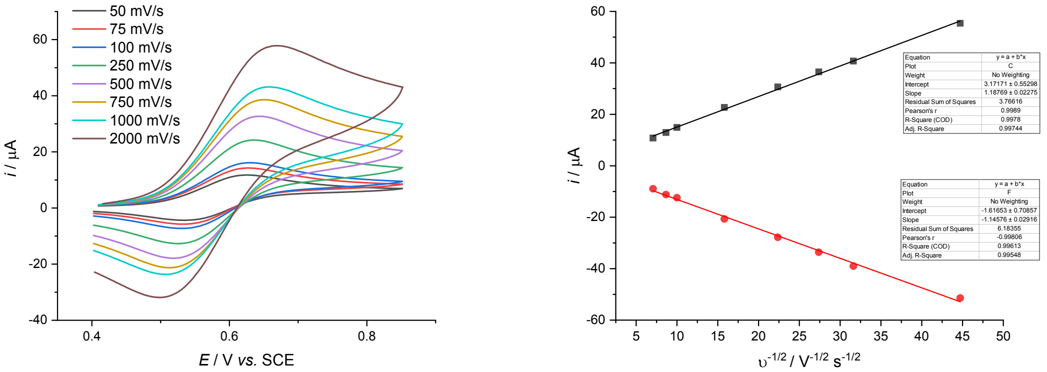

Figure S20. Scan rate studies at different scan rates from 0.05 to 2 V·s^{-1}of 1st oxidation of complex **2** (left) and scan rate analysis (right) in anhydrous acetonitrile with 0.1 M of $TBAPF_6$ at 25°C, under Ar atmosphere.

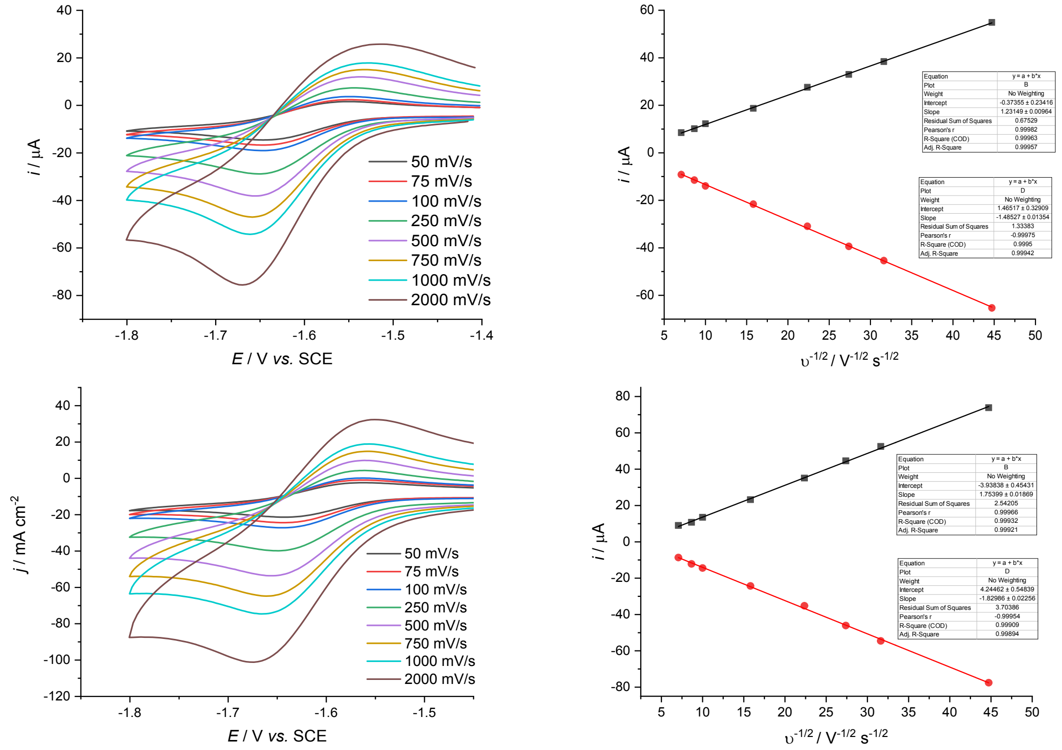

Figure S21. Scan rate studies at different scan rates from 0.05 to 2 V·s^{-1}of 1st reduction process and scan rate analysis of complex **3** (top) and **4** (bottom) in anhydrous acetonitrile with 0.1 M of TBAPF$_6$ at 25°C, under N$_2$ atmosphere recorded at different scan rates from 0.05 to 2 V/s.

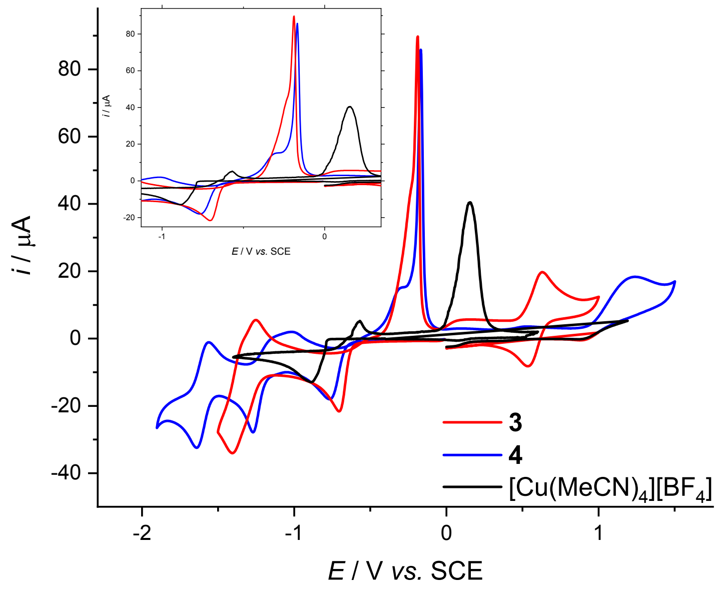

Figure S22. Cyclic voltammetries under Argon of **3**, **4** and 1 equivalent of [Cu(MeCN)$_4$][BF$_4$] in anhydrous acetonitrile with 0.1 M of TBAPF$_6$ at 25°C at 100 V·s^{-1}, [**3**] = 1 mM; [**4**] = 1 mM; [[Cu(MeCN)$_4$][BF$_4$]] = 1 mM.

Table S1. Potential values for the first cathodic event under Argon of complexes **3**, **4** and [Cu(MeCN)$_4$][BF$_4$] in anhydrous acetonitrile with 0.1 M of TBAPF$_6$ at 25°C at 100 mV·s^{-1}.

**E (V) *vs.* SCE**	**FeCu**	**NiCu**	**Cu(MeCN)$_4$BF4**
1st reduction	−0.7 V	−0.76 V	−0.9 V

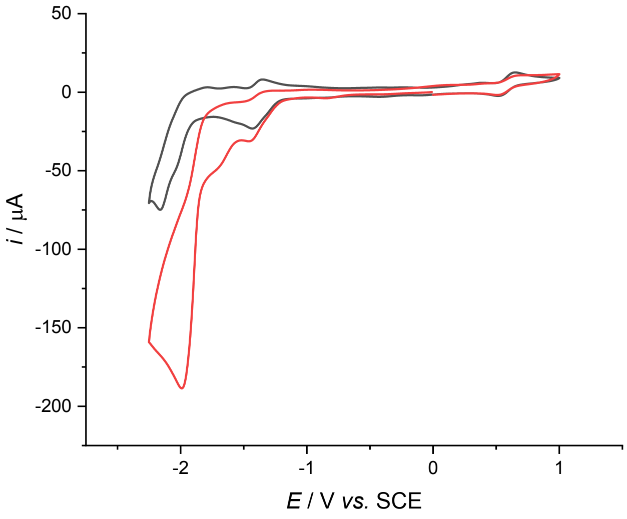

Figure S23. Cyclic Voltammetry of monometallic complex **1** [1mM] recorded in anhydrous acetonitrile with 0.1 M of TBAPF$_6$ at 25°C, under Argon (black) and CO_2 (red) at 100 mV/s.

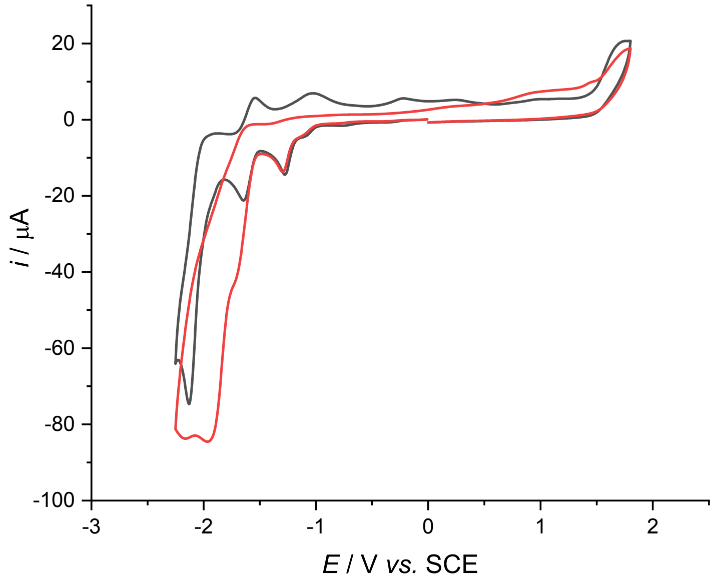

Figure S24. Cyclic Voltammetry of monometallic complex **2** [1 mM] recorded in anhydrous acetonitrile with 0.1 M of $TBAPF_6$ at 25°C, under Argon (black) and CO_2 (red) at 100 mV/s.

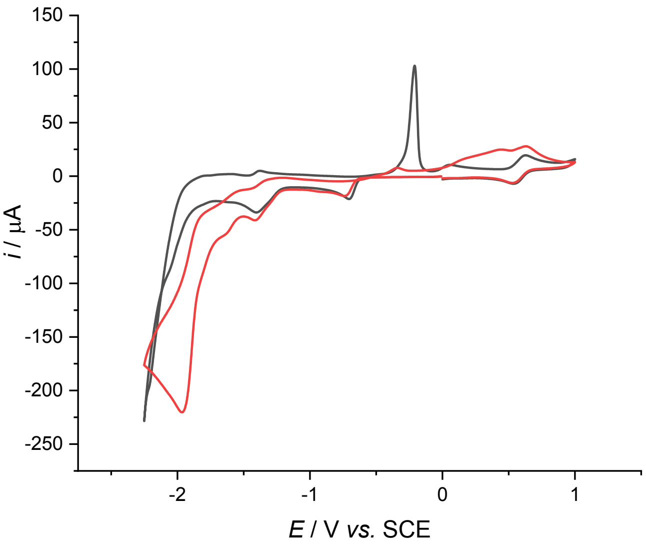

Figure S25. Cyclic Voltammetry of heterobimetallic complex **3** [1mM] recorded in anhydrous acetonitrile with 0.1 M of $TBAPF_6$ at 25°C, under Argon (black) and CO_2 (red) at 100 mV/s.

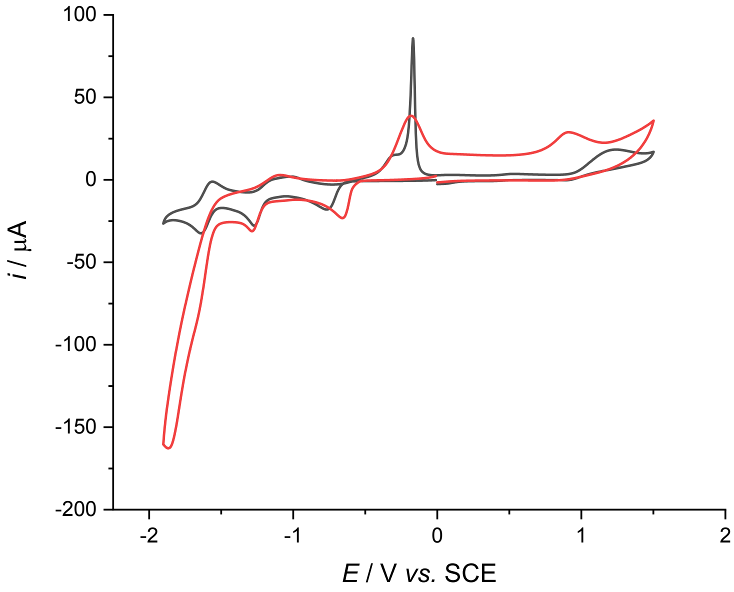

Figure S26. Cyclic Voltammetry of heterobimetallic complex **4** [1mM] recorded in in anhydrous acetonitrile with 0.1 M of $TBAPF_6$ at 25°C, under Argon (black) and CO_2 (red) at 100 mV/s.

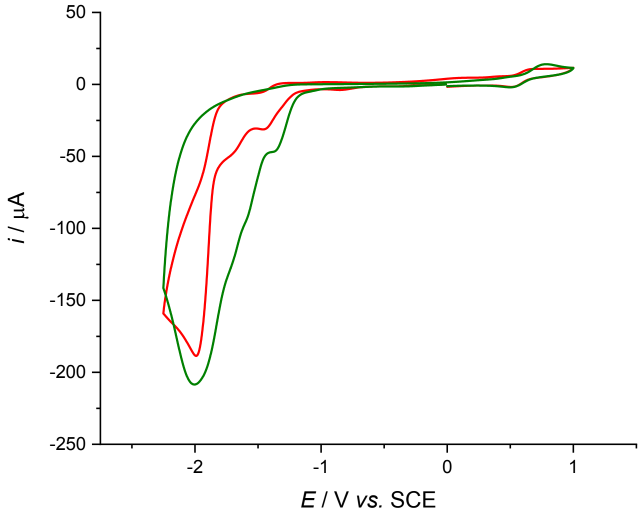

Figure S27. CV of the monometallic complex **1** [1 mM] in anhydrous acetonitrile with 0.1 M of $TBAPF_6$ at 25°C, under CO_2 (red line) and in presence of 100 μL of TFE additions (green line).

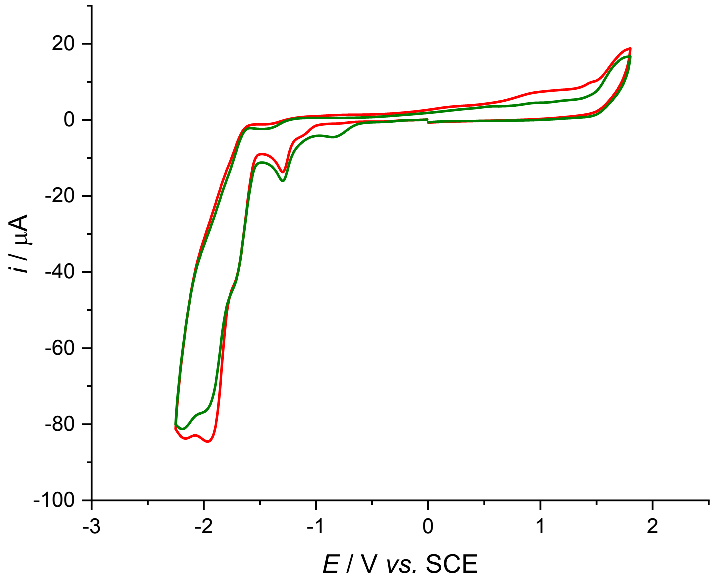

Figure S28. CV of the monometallic complex **2** [1 mM] in anhydrous acetonitrile with 0.1 M of $TBAPF_6$ at 25°C, under CO_2 (red line) and in presence of 100 µL of TFE additions (green line).

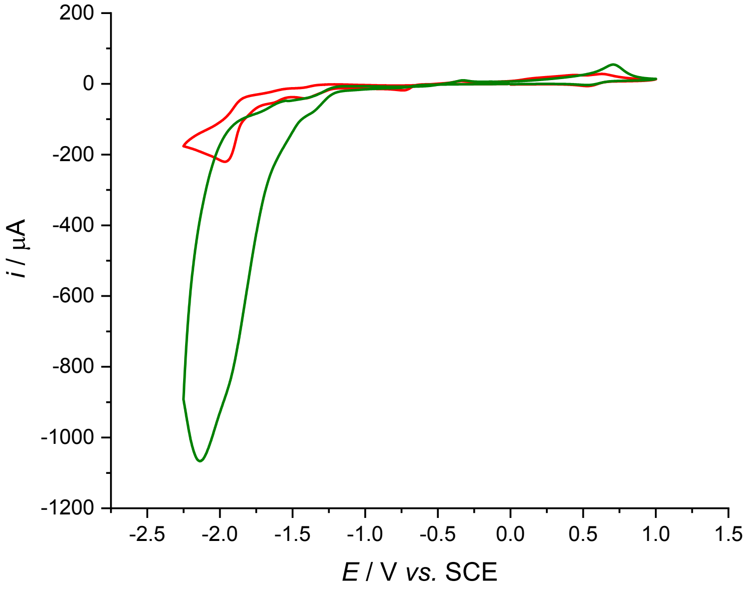

Figure S29. CV of bimetallic complex **3** [1 mM] in anhydrous acetonitrile with 0.1 M of $TBAPF_6$ at 25°C, under CO_2 (red line) and in presence of 100 µL of TFE additions (green line).

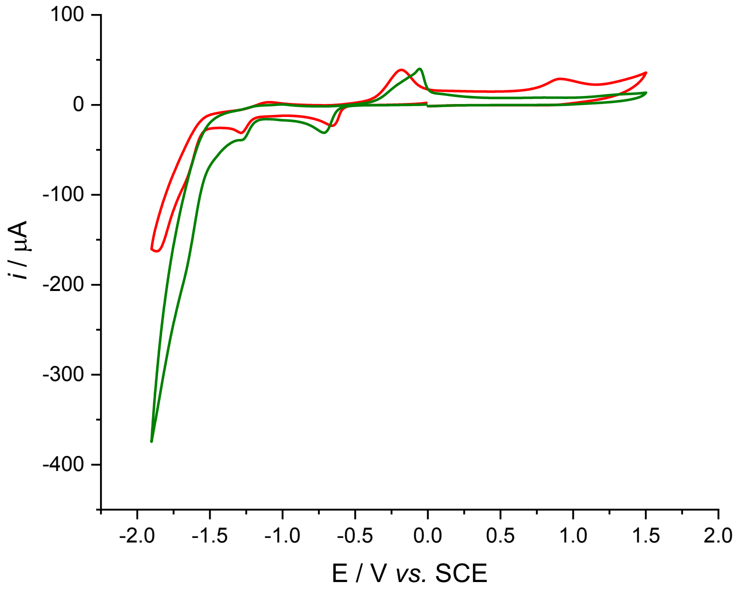

Figure S30. CV of bimetallic complex **4** [1 mM] in anhydrous acetonitrile with 0.1 M of $TBAPF_6$ at 25°C, under CO_2 (red line) and in presence of 100 µL of TFE additions (green line).

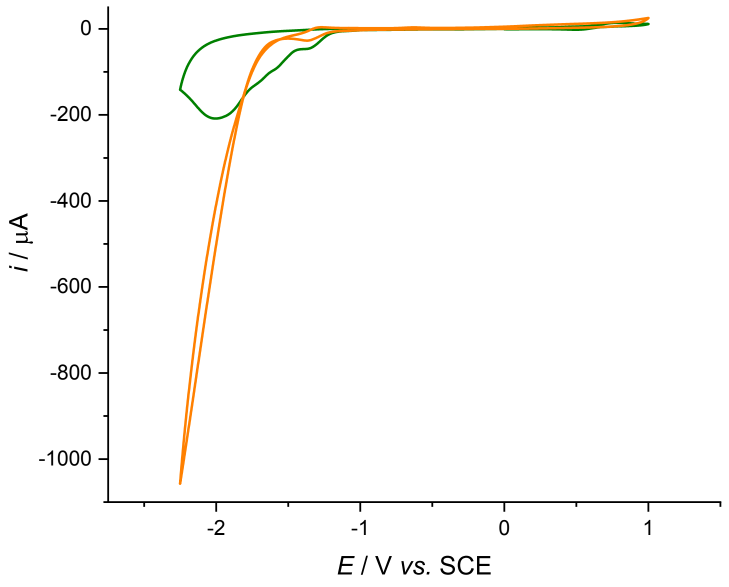

Figure S31. CV of the monometallic complex **1** [1 mM] in anhydrous acetonitrile with 0.1 M of $TBAPF_6$ at 25°C, under Ar (orange line) and under CO_2 (green line), both in presence of 100 µL of TFE.

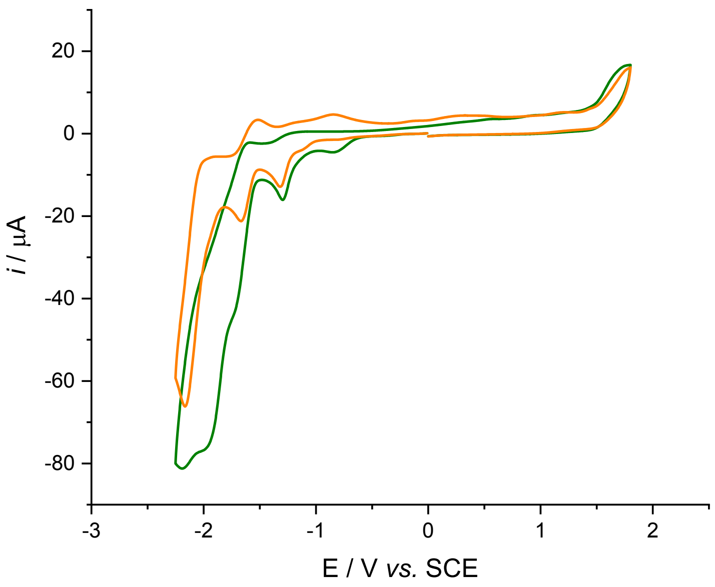

Figure S32. CV of the monometallic complex **2** [1 mM] in anhydrous acetonitrile with 0.1 M of $TBAPF_6$ at 25°C, under Ar (orange line) and under CO_2 (green line), both in presence of 100 µL of TFE.

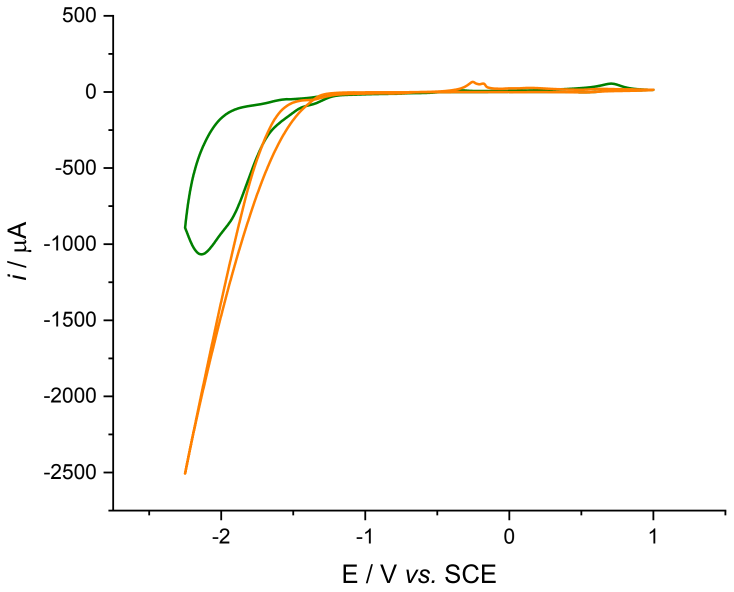

Figure S33. CV of the bimetallic complex **3** [1 mM] in anhydrous acetonitrile with 0.1 M of $TBAPF_6$ at 25°C, under Ar (orange line) and under CO_2 (green line), both in presence of 100 µL of TFE.

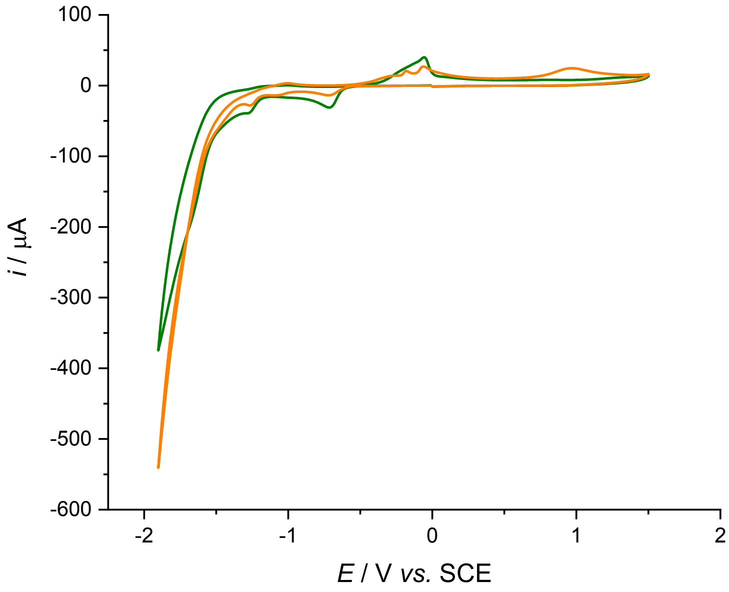

Figure S34. CV of the bimetallic complex **4** [1 mM] in anhydrous acetonitrile with 0.1 M of $TBAPF_6$ at 25°C, under Ar (orange line) and under CO_2 (green line), both in presence of 100 μL of TFE.

UV–visible spectro−electrochemical experiments and comparison with chemical reduction

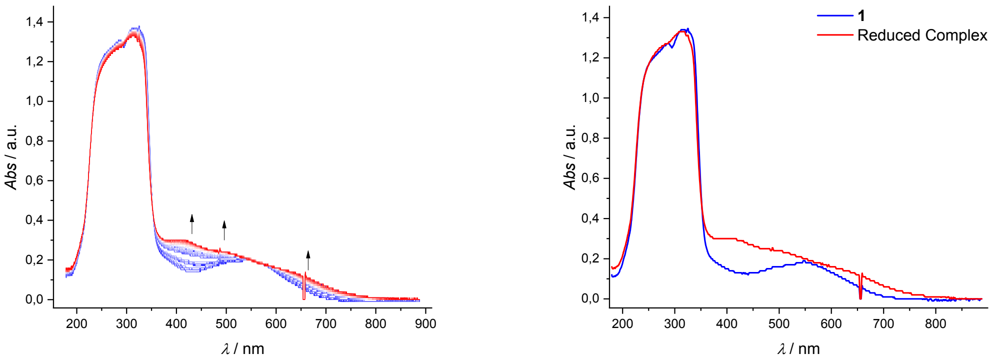

Figure S35. UV–visible spectra of 1 mM of complex **1** before (blue) and after (red) controlled potential electrolysis at –1.7 V. Spectra are recorded in anhydrous acetonitrile with 0.1 M of $TBAPF_6$ at 25°C, under Ar.

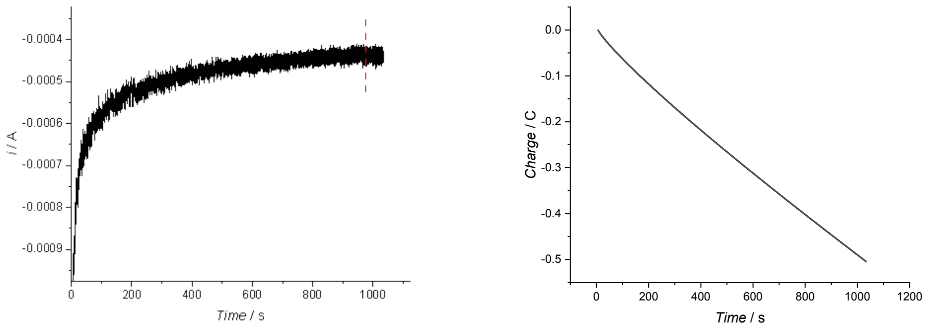

Figure S36. Controlled potential electrolysis of complex **1** at – 1.7 V *vs.* SCE in anhydrous acetonitrile with 0.1 M of $TBAPF_6$ at 25°C, under Ar. Red dashed line indicates the time needed (977 seconds) to transfer 2 mols of electrons (0.48 C) to the species under study. Therefore, the UV–vis spectrum in red presented on Figure S35 – right, was obtained at the time indicated by the red dashed line.

- Chemical reduction of complex **1** with 2 equivalents of decamethylcobaltocene ($CoCp^*_2$).

Inside the glove box, to a MeCN solution of complex **1** (1 mM), 2 equivalents of recrystallized commercial $CoCp^*_2$ were added. This was performed by addition of a freshly prepared solution of $CoCp^*_2$. The reaction was followed by UV–vis spectroscopy. See Figure S37.

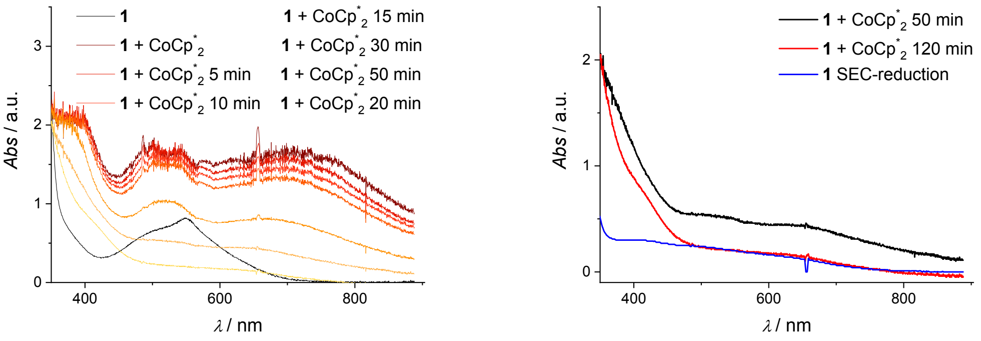

Figure S37. Left: UV–vis spectra stacking showing the evolution of reacting complex **1** (1 mM) with 2 equivalents of $CoCp^*_2$, in MeCN solutions. Right: Comparison of UV–vis spectrum obtained by electrochemical means (blue line) with UV–vis spectra obtained by chemical reduction (black and red lines).

Although similar UV–vis spectra are obtained from electrochemical and chemical reduction (two electrons) of complex **1** at certain periods of time. The fast evolution of the species generated, does not allow its isolation and therefore its full characterization.

Calculation of number of electrons

We considered that 2 mols of electrons are necessary to promote the first reduction process observed in cyclic voltammetry for complex **1**. Thus, we performed a controlled potential electrolysis coupled to UV–vis spectroelectrochemical analysis, to characterize the redox event that was taking place during the reduction of complex **1**.

Hence, considering the redox process:

$$\mathbf{1} + 2e^{-} \rightleftharpoons \mathbf{1red}$$

We calculated the necessary charge to reduce 2 mols of electrons of **1** using the following equation [REF]:

$$m_{\mathbf{1}} = \frac{Q}{nF}$$

where m_1 is the number of moles of the initial species, Q is the charge passed (in coulombs, C), n is the number of electrons involved in the process and F is the Faraday constant (96485 C mol^{-1}).

During the experiment, $3x10^{-6}$ mols of **1** were used, thus theoretically 0.481C are necessary to promote the 2–electron reduction process. The UV–vis spectra of the reduced species shown in Figure S35 in red color, was obtained after passing 0.481 C during the CPE experiment. The red dashed line in Figure S36 indicate the passing of passing 0.481 C.

Controlled Potential Electrolysis (CPE) Studies

Controlled potential electrolysis were conducted using an AUTOLAB PGSTAT302 (Metrohm). Preparative scale controlled potential electrolysis (CPE) experiments were performed in an electrolysis cell with a working compartment (4.5 mL liquid volume) and counter compartment (2 mL liquid volume) separated by an ultrafine glass frit, the total volume of the sealed cell is 25 mL, all CPEs were performed at +20 °C. A 2 cm^2 glassy carbon plate was used as the working electrode, a platinum grid was used as the auxiliary electrode, and a Ag/Ag^+ in a tipped glass tube filled with electrolyte ($TBAPF_6$, 0.5 M in CH_3CN) was used as a reference electrode. Both compartments were sealed to be gastight. A second glassy carbon electrode (0.03 cm^2 area) was added in the working compartment to perform CV scan before and after the CPE measurement. The working compartment was sparged with CO_2 for 10 min before adding the solutions. The electrolyte solution was constantly stirred during the CPE experiment with a 1 cm stirring bar. No iR compensation was applied. The electrolysis experiments were then conducted at constant potential for the specified amount of time (3 h). After this period, the headspace of the cell was immediately analysed by gas chromatography (GC).

Table S2. Results from the CPEs performed. Potentials *vs.* Ag/Ag^{+}.

Entry	Cat.	FE%–H_2 (μmol)	FE%–CO (μmol)	FE%–CH_4 (μmol)	[Cat] mM	Potential V	Table SX (Page SX)
1	**1**	1 (0,3)	19 (9)	0 (0)	2	– 1,7	S3 (S37)
2	**2**	0 (0)	0 (0,5)	2 (6)	2	– 1,7	S4 (S39)
3	**3**	6 (16)	5 (13)	17 (22)	2	– 1,7	S5 (S41)
4	**4**	0	0	7 (2)	2	– 1,7	S 6 (S43)
5	**3**	11 (27)	2 (5)	10 (28)	2	– 1,8	S7 (S45)
6	**1**	2 (0,3)	9 (2)	0 (0)	0,5	– 1,7	S8 (S47)

Applied Potential (V *vs.* Ag/Ag^+)	−1.7		
Charged Passed (C)	9.5		
Quantity of catalyst (mol)	$9\cdot10^{-6}$		
Time (s)	10800		
Gasses formed	H_2	CO	CH_4
Quantity (mol)	$0.3\cdot10^{-6}$	$8.9\cdot10^{-6}$	0
Faradaic Efficiency (%)	1	19	0

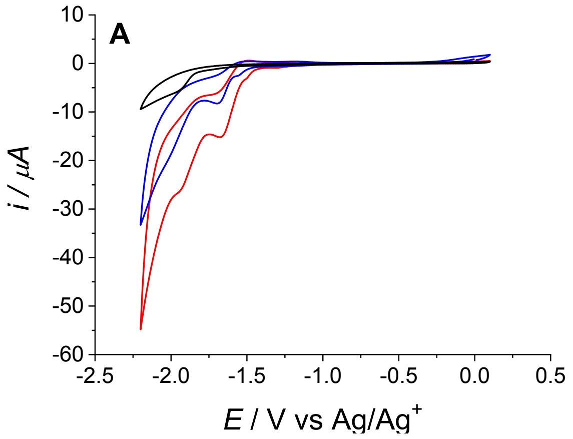

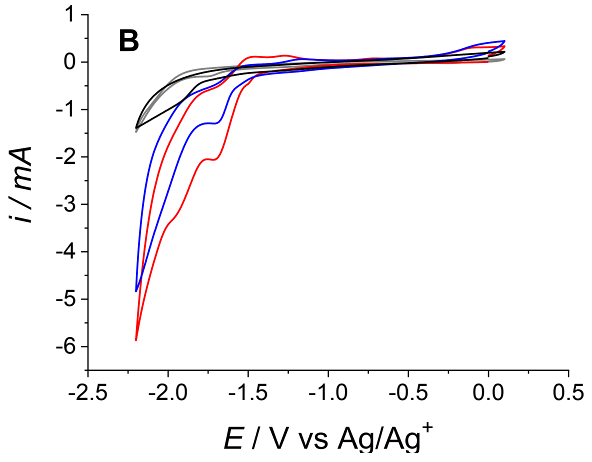

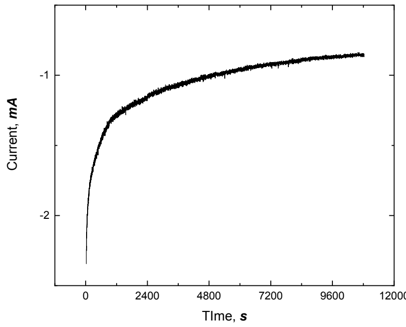

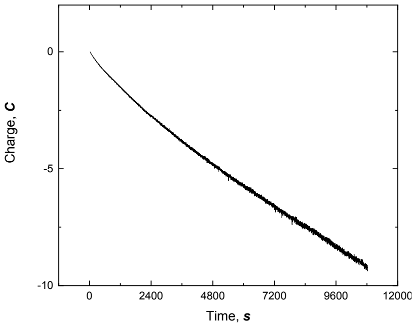

Table S3. Bottom) Current and charge overtime during CPE experiments of complex **1** (2 mM) in anhydrous MeCN under CO_2 with 10% V/V of CF_3CH_2OH. A) CVs of complex **1** (2 mM) under CO_2 in anhydrous MeCN with 10% V/V of CF_3CH_2OH before CPE (red) and after CPE (blue). Glassy carbon electrode of 0.03 cm^2 area, scan rate of 0.1 $V \cdot s^{-1}$. B) CVs of a saturated solution of CO_2 in anhydrous MeCN with 10% V/V of CF_3CH_2OH (grey) of complex **1** (2 mM) before CPE (red) and after CPE (blue) with the same glassy carbon plate in a solution without catalyst with 10% V/V of CF_3CH_2OH in MeCN under CO_2 (black). Glassy carbon plate of 2 cm^2 area, scan rate of 0.1 $V \cdot s^{-1}$.

Applied Potential (V *vs.* Ag/Ag^+)	−1.7		
Charged Passed (C)	1.9		
Quantity of catalyst (mol)	$9 \cdot 10^{-6}$		
Time (s)	10800		
Gasses formed	H_2	CO	CH_4
Quantity (mol)	0	$0.5 \cdot 10^{-6}$	$5.6 \cdot 10^{-6}$
Faradaic Efficiency (%)	0	5	9

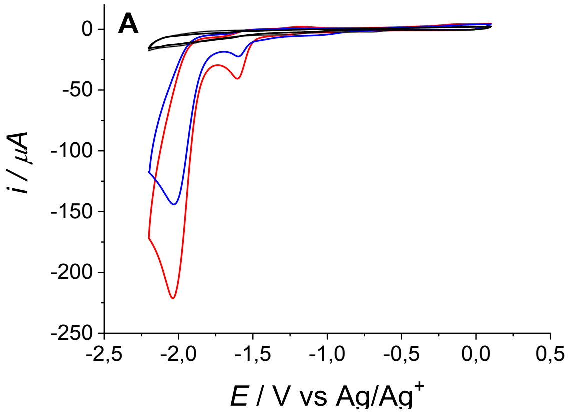

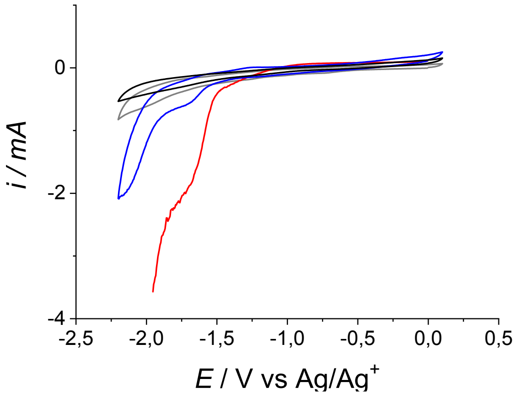

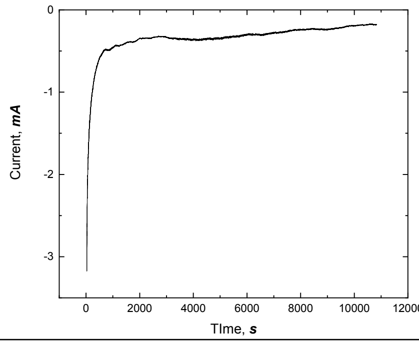

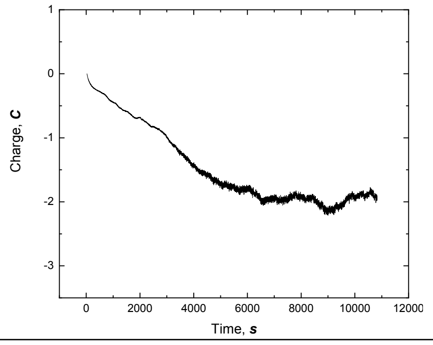

Table S4. Bottom) Current and charge overtime during CPE experiments of complex **2** (2 mM) in anhydrous MeCN under CO_2 with 10% V/V of CF_3CH_2OH. A) CVs of a saturated solution of CO_2 in anhydrous MeCN with 10% V/V of CF_3CH_2OH (grey), of complex **2** (2 mM) under CO_2 in anhydrous MeCN with 10% V/V of CF_3CH_2OH before CPE (red) and after CPE (blue) with the same glassy carbon of 0.03 cm^2 area in a solution without catalyst with 10% V/V of CF_3CH_2OH in MeCN under CO_2 (black). Scan rate of 0.1 $V\cdot s^{-1}$. B) CVs of a saturated solution of CO_2 in anhydrous MeCN with 10% V/V of CF_3CH_2OH (grey) of complex **2** (2 mM) before CPE (red–LSV) and after CPE, (blue) with the same glassy carbon plate in a solution without catalyst with 10% V/V of CF_3CH_2OH in MeCN under CO_2 (black). Glassy carbon plate of 2 cm^2 area, scan rate of 0.1 $V\cdot s^{-1}$.

Applied Potential (V *vs.* Ag/Ag^+)	−1.7		
Charged Passed (C)	52		
Quantity of catalyst (mol)	$9\cdot10^{-6}$		
Time (s)	10800		
Gasses formed	H_2	CO	CH_4
Quantity (mol)	$15.8\cdot10^{-6}$	$12.9\cdot10^{-6}$	$21.9\cdot10^{-6}$
Faradaic Efficiency (%)	6	5	33

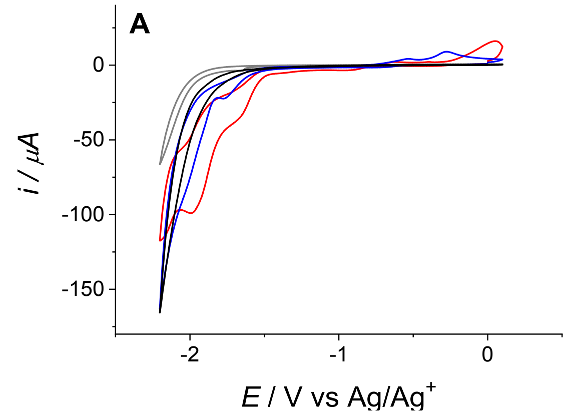

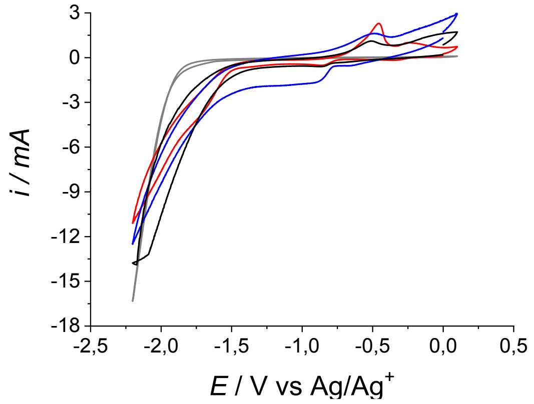

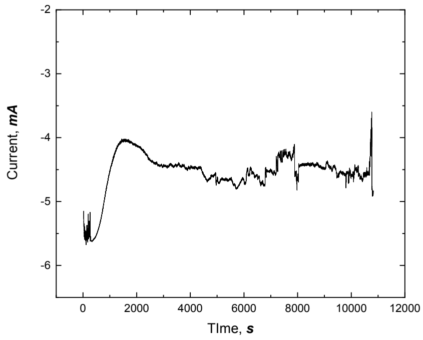

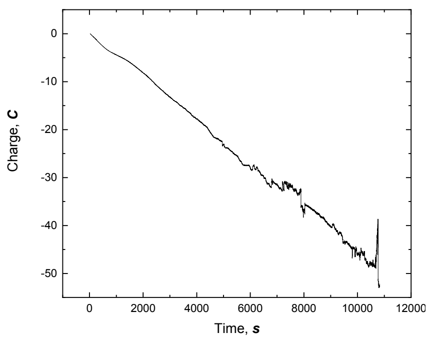

Table S5. Bottom) Current and charge overtime during CPE experiments of complex **3** (2 mM) in anhydrous MeCN under CO_2 with 10% V/V of CF_3CH_2OH. A) CVs of a saturated solution of CO_2 in anhydrous MeCN with 10% V/V of CF_3CH_2OH (grey), of complex **3** (2 mM) under CO_2 in anhydrous MeCN with 10% V/V of CF_3CH_2OH before CPE (red) and after CPE (blue) with the same glassy carbon of 0.03 cm^2 area in a solution without catalyst with 10% V/V of CF_3CH_2OH in MeCN under CO_2 (black). Scan rate of 0.1 $V \cdot s^{-1}$. B) CVs of a saturated solution of CO_2 in anhydrous MeCN with 10% V/V of CF_3CH_2OH (grey) of complex **3** (2 mM) before CPE (red) and after CPE (blue) with the same glassy carbon plate in a solution without catalyst with 10% V/V of CF_3CH_2OH in MeCN under CO_2 (black). Glassy carbon plate of 2 cm^2 area, scan rate of 0.1 $V \cdot s^{-1}$.

Applied Potential (V *vs.* Ag/Ag^+)	−1.7		
Charged Passed (C)	12.18		
Quantity of catalyst (mol)	$9 \cdot 10^{-6}$		
Time (s)	10800		
Gasses formed	H_2	CO	CH_4
Quantity (mol)	0	0	$2.1 \cdot 10^{-6}$
Faradaic Efficiency (%)	0	0	14

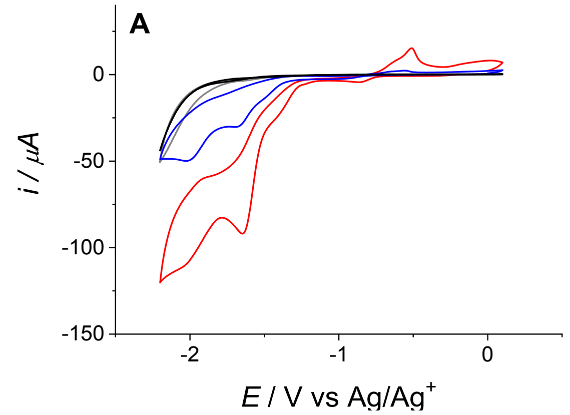

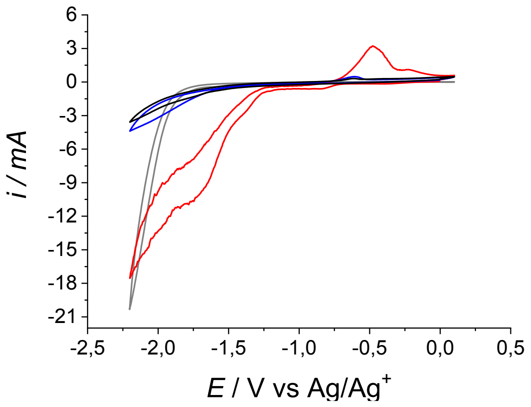

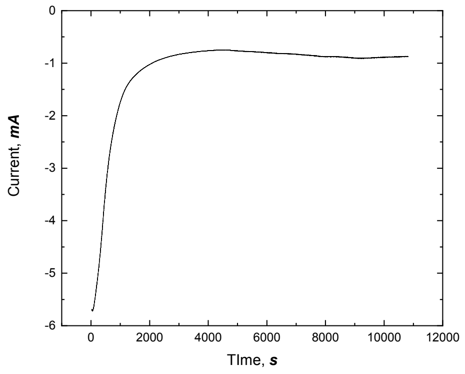

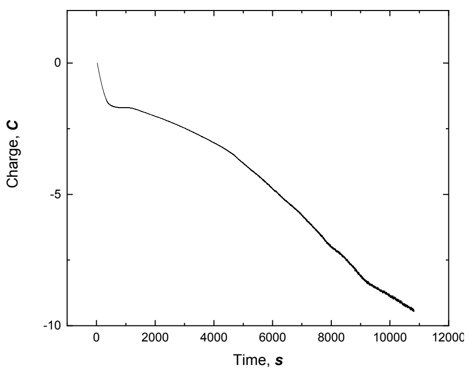

Table S6. Bottom) Current and charge overtime during CPE experiments of complex **4** (2 mM) in anhydrous MeCN under CO_2 with 10% V/V of CF_3CH_2OH. A) CVs of a saturated solution of CO_2 in anhydrous MeCN with 10% V/V of CF_3CH_2OH (grey), of complex **4** (2 mM) under CO_2 in anhydrous MeCN with 10% V/V of CF_3CH_2OH before CPE (red) and after CPE (blue) with the same glassy carbon of 0.03 cm^2 area in a solution without catalyst with 10% V/V of CF_3CH_2OH in MeCN under CO_2 (black). Scan rate of 0.1 $V{\cdot}s^{-1}$. B) CVs of a saturated solution of CO_2 in anhydrous MeCN with 10% V/V of CF_3CH_2OH (grey) of complex **4** (2 mM) before CPE (red) and after CPE (blue) with the same glassy carbon plate in a solution without catalyst with 10% V/V of CF_3CH_2OH in MeCN under CO_2 (black). Glassy carbon plate of 2 cm^2 area, scan rate of 0.1 $V{\cdot}s^{-1}$.

Applied Potential (V *vs.* Ag/Ag^{+})	−1.8		
Charged Passed (C)	49.94		
Quantity of catalyst (mol)	$9·10^{-6}$		
Time (s)	10800		
Gasses formed	H_2	CO	CH_4
Quantity (mol)	$27.2·10^{-6}$	$4.8·10^{-6}$	$27.6·10^{-6}$
Faradaic Efficiency (%)	11	2	43

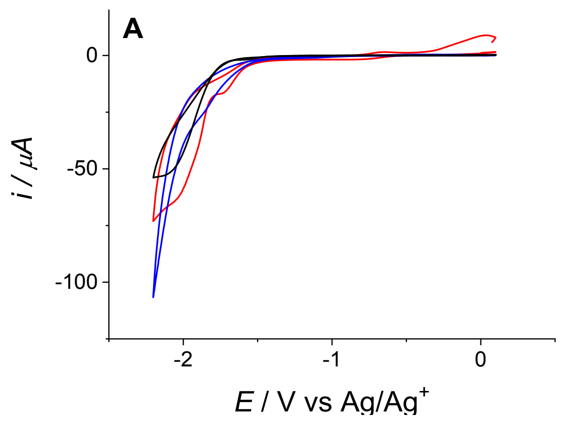

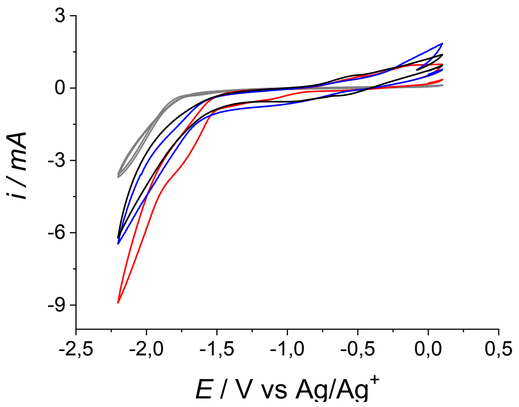

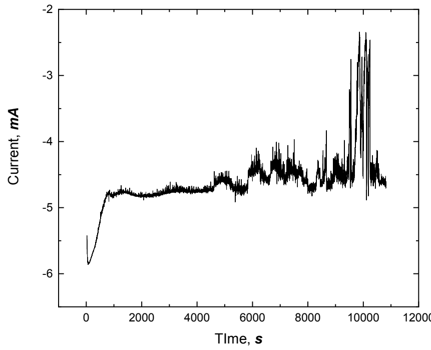

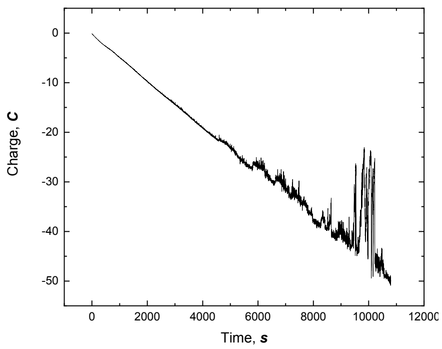

Table S7. Bottom) Current and charge overtime during CPE experiments of complex **3** (2 mM) in anhydrous MeCN under CO_2 with 10% V/V of CF_3CH_2OH. A) CVs of a saturated solution of CO_2 in anhydrous MeCN with 10% V/V of CF_3CH_2OH (grey), of complex **3** (2 mM) under CO_2 in anhydrous MeCN with 10% V/V of CF_3CH_2OH before CPE (red) and after CPE (blue) with the same glassy carbon of 0.03 cm^2 area in a solution without catalyst with 10% V/V of CF_3CH_2OH in MeCN under CO_2 (black). Scan rate of 0.1 $V\cdot s^{-1}$. B) CVs of a saturated solution of CO_2 in anhydrous MeCN with 10% V/V of CF_3CH_2OH (grey) of complex **3** (2 mM) before CPE (red) and after CPE (blue) with the same glassy carbon plate in a solution without catalyst with 10% V/V of CF_3CH_2OH in MeCN under CO_2 (black). Glassy carbon plate of 2 cm^2 area, scan rate of 0.1 $V\cdot s^{-1}$.

Applied Potential (V *vs.* Ag/Ag$^+$)	−1.7		
Charged Passed (C)	3.34		
Quantity of catalyst (mol)	$2.3\cdot10^{-6}$		
Time (s)	9467		
Gasses formed	H_2	CO	CH_4
Quantity (mol)	$0.3\cdot10^{-6}$	$1.5\cdot10^{-6}$	0
Faradaic Efficiency (%)	2	9	0

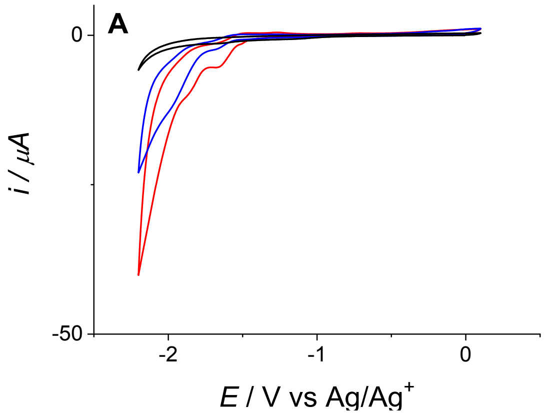

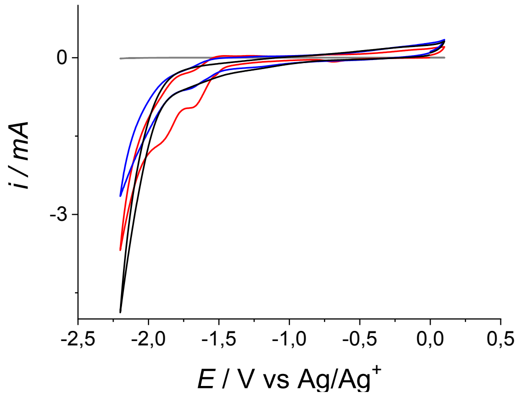

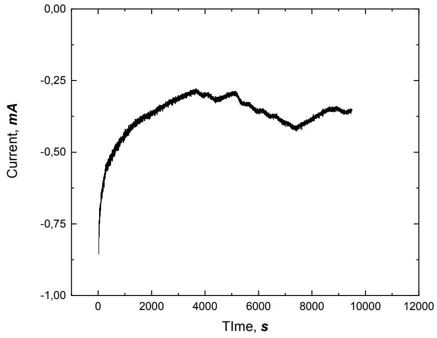

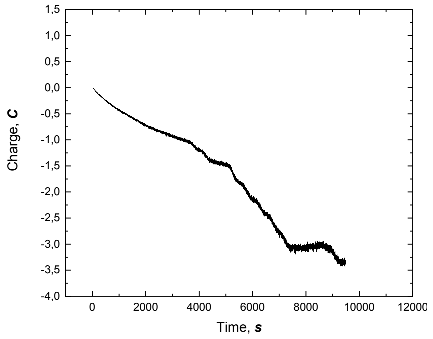

Table S8. Bottom) Current and charge overtime during CPE experiments of complex **1** (0.5 mM) in anhydrous MeCN under CO_2 with 10% V/V of CF_3CH_2OH. A) CVs of a saturated solution of CO_2 in anhydrous MeCN with 10% V/V of CF_3CH_2OH (grey), of complex **1** (0.5 mM) under CO_2 in anhydrous MeCN with 10% V/V of CF_3CH_2OH before CPE (red) and after CPE (blue) with the same glassy carbon of 0.03 cm^2 area in a solution without catalyst with 10% V/V of CF_3CH_2OH in MeCN under CO_2 (black). Scan rate of 0.1 $V \cdot s^{-1}$. B) CVs of a saturated solution of CO_2 in anhydrous MeCN with 10% V/V of CF_3CH_2OH (grey) of complex **1** (0.5 mM) before CPE (red) and after CPE (blue) with the same glassy carbon plate in a solution without catalyst with 10% V/V of CF_3CH_2OH in MeCN under CO_2 (black). Glassy carbon plate of 2 cm^2 area, scan rate of 0.1 $V \cdot s^{-1}$.

Applied Potential (V *vs.* Ag/Ag$^+$)	−1.7
Charged Passed (C)	23.5
Quantity of catalyst (mol)	$9 \cdot 10^{-6}$
Time (s)	10800

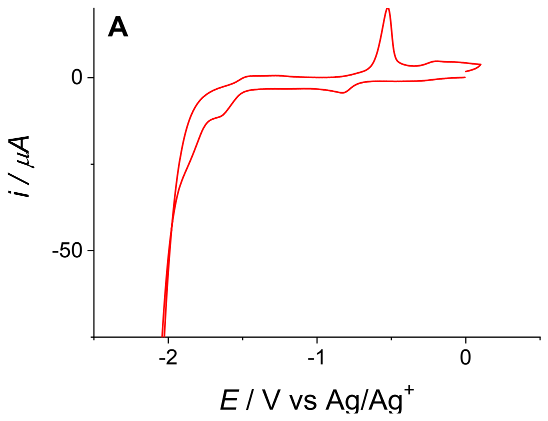

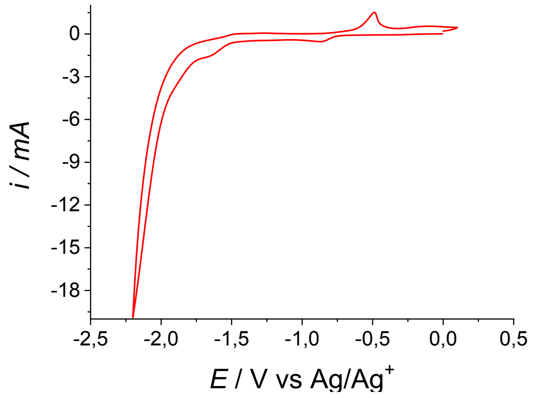

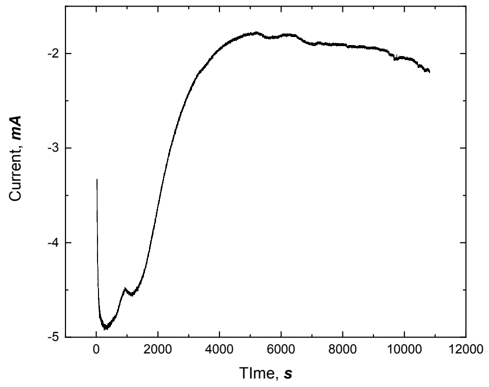

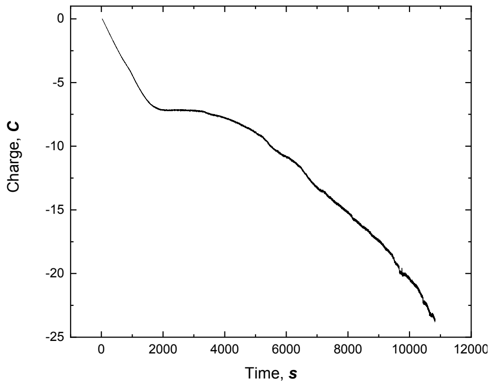

Table S9. Bottom) Current and charge overtime during CPE experiments of complex **3** (2 mM) in anhydrous MeCN under $^{13}CO_2$ with 10% V/V of CF_3CH_2OH. A) CVs of complex **3** (2 mM) under $^{13}CO_2$ in anhydrous MeCN with 10% V/V of CF_3CH_2OH before

CPE (red). Scan rate of 0.1 V·s^{-1}. B) CVs of complex **3** (2 mM) under $^{13}CO_2$ in anhydrous MeCN with 10% V/V of CF_3CH_2OH before CPE (red) with the glassy carbon. Glassy carbon plate of 2 cm² area, scan rate of 0.1 V·s^{-1}.

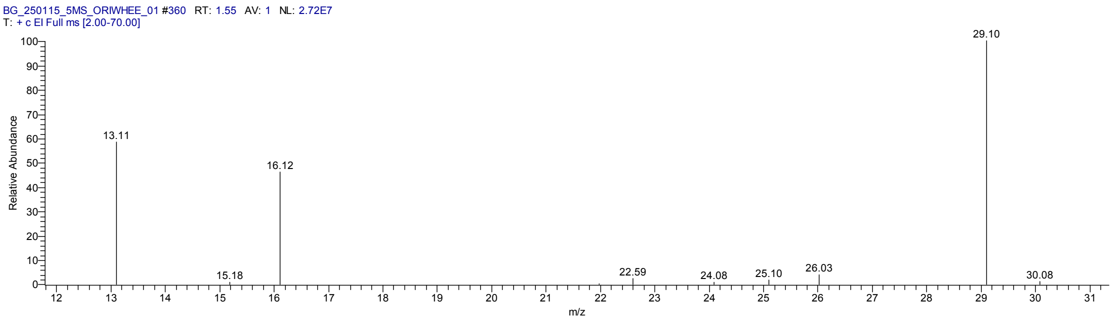

Figure S38. Mass spectra of the headspace corresponding to the previous CPE undergone under labelled $^{13}CO_2$ (Table S9). $^{12}CH_4$ (m/z = 16), ^{13}CO (m/z = 29), $^{13}CH_4$ (m/z = 17) non detected.

XPS Analysis

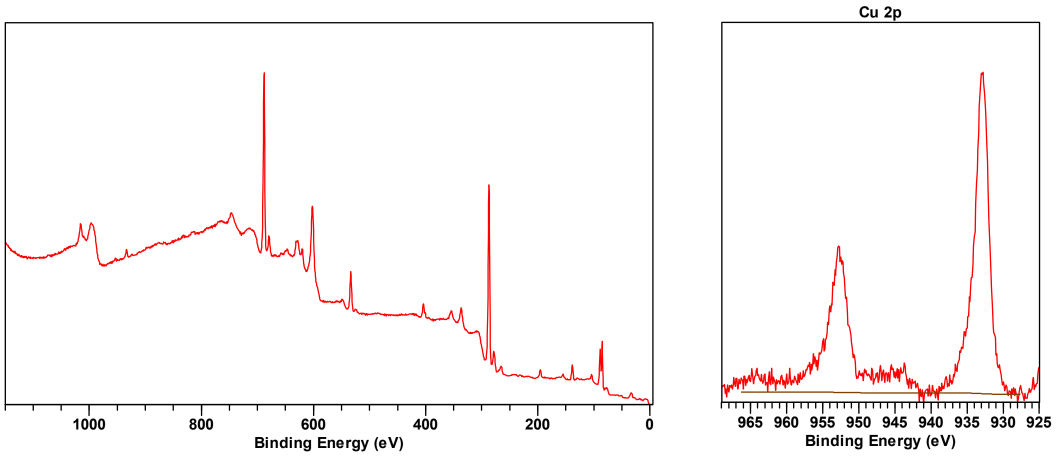

Figure S39. Left, XPS spectra. Right, Cu 2p energy level at a glassy carbon plate after 3 h CPE experiment using solutions of complex **3**. Conditions during deposition: saturated solution of CO_2 in anhydrous MeCN with 10% V/V of CF_3CH_2OH of complex **3** (2 mM).

Photocatalytic CO2RR experiments

Table S10. TON of produced H_2 and CO from CO2RR photocatalytic experiments using as catalyst complexes **1 – 4** all under same conditions: Catalyst [5 µM], with BIH as ED [25 µM], $Ru(phen)_3Cl_2$ as PS [0.2 mM] and TFE 10% V/V as H^+D after 24 hr of irradiation.

Complex	TON, H2	TON, CO
**1**	12 (±20%)	131 131 (±2%)
**2**	0.32	0.74
**3**	20.2 (±12%)	152 (±30%)
**4**	0.29	0.35
**without catalyst**	0	0

References:

(1) Alzakhem, N.; Bischof, C.; Seitz, M. Dependence of the Photophysical Properties on the Number of 2,2′–Bipyridine Units in a Series of Luminescent Europium and Terbium Cryptates. *Inorg. Chem.* **2012**, *51* (17), 9343–9349. https://doi.org/10.1021/ic3010568.

(2) Tretbar, M.; Stark, C. B. W. Large–Scale Synthesis of Symmetric Tetramine Ligands by Using a Modular Double Reductive Amination Approach. *European Journal of Organic Chemistry* **2017**, *2017* (46), 6942–6946. https://doi.org/10.1002/ejoc.201700772.

(3) Bohn, A.; Moreno, J. J.; Thuéry, P.; Robert, M.; Rivada–Wheelaghan, O. Electrocatalytic CO2 Reduction with a Binuclear Bis–Terpyridine Pyrazole–Bridged Cobalt Complex**. *Chemistry – A European Journal* **2023**, *29* (9), e202202361. https://doi.org/10.1002/chem.202202361.
